# Use of alcohol, tobacco, cannabis, and other substances during the first wave of the SARS-CoV-2 pandemic in Europe: a survey on 36,000 European substance users

**DOI:** 10.1186/s13011-021-00373-y

**Published:** 2021-04-26

**Authors:** Jakob Manthey, Carolin Kilian, Sinclair Carr, Miroslav Bartak, Kim Bloomfield, Fleur Braddick, Antoni Gual, Maria Neufeld, Amy O’Donnell, Benjamin Petruzelka, Vladimir Rogalewicz, Ingeborg Rossow, Bernd Schulte, Jürgen Rehm

**Affiliations:** 1 Institute of Clinical Psychology and Psychotherapy, Technische Universität Dresden, Chemnitzer Str. 46, 01187 Dresden, Germany; 2 Center for Interdisciplinary Addiction Research (ZIS), Department of Psychiatry and Psychotherapy, University Medical Center Hamburg-Eppendorf (UKE), Martinistraße 52, 20246 Hamburg, Germany; 3 Department of Psychiatry, Medical Faculty, University of Leipzig, Semmelweisstraße 10, 04103 Leipzig, Germany; 4 First Faculty of Medicine and General Teaching Hospital Prague, Department of Addiction, Charles University, Apolinarska 4, 128 00 Prague 2, Czech Republic; 5 Centre for Alcohol and Drug Research, Aarhus University, Tuborgvej 160, 2400 Copenhagen, NV Denmark; 6 Institute of Biometry and Clinical Epidemiology, Charité – Universitätsmedizin Berlin, Charitéplatz 1, 10117 Berlin, Germany; 7 Health Promotion Department of Public Health, University of Southern Denmark, Niels Bohrs Vej 9, DK-6700 Esbjerg, Denmark; 8 Alcohol Research Group, Public Health Institute, 6001 Shellmound Street, Suite 450, Emeryville, CA 94608 USA; 9 Clínic Foundation for Biomedical Research (FCRB), 08036 Barcelona, Spain; 10 Clinical Addictions Research Group (GRAC-GRE) Psychiatry Department, Neurosciences Institute, Hospital Clínic, University of Barcelona, 08036 Barcelona, Spain; 11 Institut d’Investigacions Biomèdiques August Pi i Sunyer (IDIBAPS), 08036 Barcelona, Spain; 12 WHO European Office for the Prevention and Control of Noncommunicable Diseases (NCD Office) 9 Leontyevsky Pereulok, Moscow, Russian Federation 125009; 13 Institute for Mental Health Policy Research, Centre for Addiction and Mental Health, 33 Ursula Franklin Street, Toronto, Ontario M5S 2S1 Canada; 14 Population Health Sciences Institute, Newcastle University, Baddiley-Clark Building, Newcastle Upon Tyne, NE2 4AX UK; 15 Norwegian Institute of Public Health, Dept of Alcohol, Tobacco and Drugs, N-0213 Oslo, Norway; 16 Dalla Lana School of Public Health, University of Toronto, 155 College Street, Toronto, Ontario M5T 1P8 Canada; 17 Faculty of Medicine, Institute of Medical Science, University of Toronto, Medical Sciences Building, 1 King’s College Circle, Room 2374, Toronto, Ontario M5S 1A8 Canada; 18 Campbell Family Mental Health Research Institute, Centre for Addiction and Mental Health, 33 Russell Street, Toronto, Ontario M5S 3M1 Canada; 19 Department of Psychiatry, University of Toronto, 250 College Street, 8th floor, Toronto, Ontario M5T 1R8 Canada; 20 I.M. Sechenov First Moscow State Medical University (Sechenov University), Trubetskaya Street 8, b. 2, Moscow, Russian Federation 119991

**Keywords:** Alcohol, Tobacco, Cannabis, Substance use, Europe, COVID-19, Survey

## Abstract

**Background:**

SARS-CoV-2 reached Europe in early 2020 and disrupted the private and public life of its citizens, with potential implications for substance use. The objective of this study was to describe possible changes in substance use in the first months of the SARS-CoV-2 pandemic in Europe.

**Methods:**

Data were obtained from a cross-sectional online survey of 36,538 adult substance users from 21 European countries conducted between April 24 and July 22 of 2020. Self-perceived changes in substance use were measured by asking respondents whether their use had decreased (slightly or substantially), increased (slightly or substantially), or not changed during the past month. The survey covered alcohol (frequency, quantity, and heavy episodic drinking occasions), tobacco, cannabis, and other illicit drug use. Sample weighted data were descriptively analysed and compared across substances.

**Results:**

Across all countries, use of all substances remained unchanged for around half of the respondents, while the remainder reported either a decrease or increase in their substance use. For alcohol use, overall, a larger proportion of respondents indicated a decrease than those reporting an increase. In contrast, more respondents reported increases in their tobacco and cannabis use during the previous month compared to those reporting decreased use. No distinct direction of change was reported for other substance use.

**Conclusions:**

Our findings suggest changes in use of alcohol, tobacco and cannabis during the initial months of the pandemic in several European countries. This study offers initial insights into changes in substance use. Other data sources, such as sales statistics, should be used to corroborate these preliminary findings.

**Supplementary Information:**

The online version contains supplementary material available at 10.1186/s13011-021-00373-y.

## Background

For a large portion of the adult European population, the use of psychoactive substances is deeply embedded in everyday routines, social practices, and interactions with the environment [1]. In 2020, the spread of SARS-CoV-2 disrupted private and public life throughout the world, by governments implementing various physical-distancing and/or lockdown policy measures, which led to substantial changes in work, learning and leisure environments, functioning of social roles, and provision of health care. Given the scale of disruption, scholars have voiced their concerns about fears of rising alcohol [2–4] and tobacco [5, 6] use during the pandemic, although an immediate decrease in alcohol consumption has also been suggested [2]. Less attention has been devoted to the use of cannabis and other illicit substances.

For both licit and illicit substances, there is a substantial extant literature documenting common and specific determinants of changes in consumption. In particular, elevated levels of distress is a well-studied risk factor for increased substance use [2, 7]. During the initial wave of the pandemic, excess stress was associated with expected or experienced job loss and associated financial problems, increased workload, anxiety about the novel disease and its dangers. Further, closing of schools and childcare providers has led to increased domestic pressures, as sick family members or children had to be taken care of by family members. Those who have contracted SARS-CoV-2 may also experience adverse health impacts and psychological distress resulting from quarantine-associated restrictions or hospitalization. Studies conducted in the UK [8] and amongst young adults in Switzerland [9] suggest that levels of mental distress have sharply risen during lockdown. Additionally, psychological distress experienced during the pandemic has been linked to rises in tobacco smoking [10–12], alcohol consumption [11, 12], and cannabis use [11].

On the other hand, varied availability of, and access to, different substances may also have affected consumption levels. The introduction of restrictions to private and public social gatherings in many countries may have both limited the opportunities to use substances and affected the social context in which substances are often consumed [13, 14]. For licit substances, especially alcohol, availability is a known factor determining consumption. This is why the World Health Organization considers restrictions in availability to be one of the so-called ‘best buys’ in reducing alcohol use and attributable disease burden [15, 16]. During the pandemic, opportunities to purchase alcohol have declined in many countries, with outlets, kiosks and bars ordered to stay closed or to restrict their opening hours in order to reduce the number of personal contacts. However, for illicit drugs, availability is also considered to affect substance use behaviour, and some market disruptions were reported as well [17]. Preliminary data from Canada and Europe suggest a drop in availability of some substances during the pandemic, with manifest shortages in several EU countries [18]. As a result, increased prices and lower purity have been reported in Canada [19] and Europe [18, 20], particularly for illicit substances other than cannabis.

To date, evidence on the impact of SARS-CoV-2 on substance use in Europe is mixed. Analysis of household purchase data suggests increased quantities of alcohol were purchased during lockdown in UK [21] and Russia [22]. A population survey conducted in France found self-reported increases of alcohol, tobacco, and no overall change in cannabis use [11]. In Greece, however, survey findings suggest there has been a decline in alcohol use during this period [23]. Moreover, preliminary data from the European Monitoring Centre for Drugs and Drug Addiction indicate an overall decline for illicit drug use in Europe, although this may vary across substances and countries [24]. However, in this regard, differences not just among countries, but also among different regions in the given countries need to be taken into account, making the situation even more complex. To this end, very few studies have offered comparable descriptions of changes in substance use across various substances within a single country or jurisdiction (e.g., [11]), and none have done so across several countries.

In this report, we aim to investigate changes in substance use in 21 European countries during the first months of the pandemic. More specifically, we report on the self-reported changes of alcohol, tobacco, cannabis, and other illicit drug use based on survey data collected between April and July 2020.

## Methods

Individual-level data were obtained from adults (aged 18 years and over) through the cross-sectional online European Alcohol Use and COVID-19 survey conducted between April 24 and July 22 of 2020 in 21 European countries (Albania, Czechia, Denmark, Finland, France, Germany, Greece, Hungary, Iceland, Ireland, Italy, Norway, Poland, Portugal, Russia, Slovakia, Slovenia, Spain, Sweden, Ukraine, and United Kingdom).

The survey was developed in English and translated into different languages using an international network of researchers (European Study Group on Alcohol Use and COVID-19). With the help of this network, the survey was disseminated in each participating country using various strategies, with the most countries relying on social media and institutional website posts, press releases, and mailing lists (strategies per country can be viewed online [25]). Additionally, some countries employed paid social media advertisements and targeted sampling to balance skewed sampling distribution. Across all countries, a total of 40,064 respondents aged 18 to 98 (median: 41) completed the survey and provided valid data, i.e., reported sex, age, education and number of household members. In order to correct for sample biases of the convenience sample (mostly oversampling young, female and people with high education), weights were applied. Weights accounted for the national distribution of the actual population according to gender (women, men), age (18–34, 35–54, 55+ years), and education (lower, middle, high education). Population information for the most recent year available was obtained from EUROSTAT [26] or census data, where available (for details, see [27]). Sampling weights were calculated as the inverse probability for taking the survey.

Of these respondents, 82.7% reported past year alcohol use, whilst tobacco (34.2%), cannabis (13.5%), and any other illicit substance use (8.3%) was less commonly reported. For analysis purposes, our final sample was limited to respondents reporting use of at least one substance (*n* = 36,538 = 91.2% of the sample).

### Outcomes

The primary objective of the survey was to gather information on changes in alcohol use in Europe during the early months of SARS-CoV-2 (reported in detail separately) [28]. However, we included a separate section in the questionnaire with further questions to identify basic changes in the use of tobacco, cannabis and other illicit substances during the same period.

Changes in alcohol use were assessed using three drinking indicators which correspond to the AUDIT-C items [29]: i) frequency: “Did you drink alcohol less or more often in the past month?”; ii) quantity: “Did the amount of alcohol you usually drink on each drinking occasion (i.e., the volume of alcohol consumed) change in the past month?”; and iii) heavy episodic drinking (HED) occasions: “Did the frequency of drinking occasions where you drank a high amount of alcohol (i.e., 6 or more drinks) change in the past month?”

For tobacco, cannabis, and other illicit substances, changes of current use were assessed using a single item for each substance: “Did you [smoke/ consume cannabis / illicit drugs (other than cannabis)] less or more often in the past month?”. For all substances, response options allowed for differentiation between two levels of decrease (slightly less, much less) and increase (slightly more, much more), no change, and non-consumption (abstention).

### Other descriptive variables

As possible confounders to changes in substance use, we assessed socio-demographics (sex, age, education) and the 3-item AUDIT-C for past-year alcohol consumption [29]. Further measures of interest were: restrictions in day-to-day life (“In the past month, did you perceive any restrictions of public life which were implemented to contain the spread of SARS-CoV-2 (i.e., corona virus)?”); financial loss (“In the past month, have you experienced any negative consequences concerning your occupational or financial situation in relation to the spread of SARS-CoV-2 (i.e., corona virus)?”); and direct SARS-CoV-2 contact (“In the past month, have you or someone close to you (i.e., a spouse, relative or close friend) been diagnosed with the SARS-CoV-2 infection (i.e., corona virus)?”). The complete questionnaire in all languages can be accessed elsewhere [30].

### Statistical analyses

Respondents with invalid responses were removed from specific analyses. More specifically, 1.7% of respondents failed to report their alcohol use on at least one of the three AUDIT-C items and 1.2% of alcohol users failed to report changes of their consumption on at least one of the three items. For the items assessing changes in tobacco, cannabis, and other illegal drug use, 0.6, 0.8, and 0.8% of all respondents did not provide a valid response, respectively.

As a summary indicator of net substance use change, we subtracted the proportion decreasing their use from the proportion increasing their use. Using this crude indicator, positive figures denoted an overall tendency to increase substance use, whilst negative figures denoted an overall tendency to decrease substance use. The corresponding confidence intervals were bootstrapped from 10,000 random estimates sampled around each point estimate based on the weighted standard error.

Descriptive statistics were weighted by sex, age and education to account for sample biases (for details, see [27]). All analyses were conducted in R version 4.0.3 [31]. The survey data and the corresponding R code to this publication are publicly available [32, 33].

## Results

The recruited sample is summarized in Table 1 for any substance use and stratified for each substance (with overlapping samples). The unweighted sample characteristics for each country can be found in Appendix Table 1. On most other indicators, the four groups of substance users showed little variation. Alcohol use was reported by 86.9% of tobacco users, by 90.5% of cannabis users, and by 90.7% of other illegal drug users (data not shown in table).

**Table 1:** Sample characteristics by user group for the total survey sample

	Any substance users (*n* = 36,538)	Alcohol users (*n* = 35,753)	Tobacco users (*n* = 9816)	Cannabis users (*n* = 3289)	Other illegal drug users (*n* = 1961)
	% (95% CI) or mean (95% CI)				
Sex					
Female	51.6% (49.2, 54.0%)	52.1% (49.7%,54.6%)	46.6% (42.5, 50.7%)	37.8% (30.5, 45.0%)	36.0% (26.2, 45.8%)
Male	47.8% (45.0, 50.5%)	47.2% (44.4, 50.0%)	52.1% (47.8, 56.5%)	59.9% (53.8, 65.9%)	61.8% (54.0, 69.5%)
Other	0.6% (0.0, 4.4%)	0.7% (0.0, 4.5%)	1.3% (0.0, 7.6%)	2.4% (0.0, 12.1%)	2.3% (0.0, 14.2%)
Age group					
18–34	33.5% (30.4, 36.6%)	34.2% (31.0, 37.3%)	38.1% (33.1, 43.1%)	54.6% (48.0, 61.1%)	50.7% (41.8, 59.6%)
35–54	43.4% (40.7, 46.1%)	43.6% (40.9, 46.4%)	42.4% (37.9, 46.8%)	32.5% (25.0, 40.0%)	35.4% (25.6, 45.2%)
55+	23.1% (20.1, 26.1%)	22.2% (19.1, 25.2%)	19.5% (14.6, 24.4%)	12.9% (4.4, 21.4%)	13.9% (2.9, 24.9%)
Education					
Low	10.6% (6.9, 14.2%)	9.1% (5.4, 12.9%)	15.5% (9.8, 21.3%)	15.2% (5.9, 24.5%)	15.6% (3.6, 27.5%)
Middle	44.1% (41.2, 47.1%)	44.2% (41.1, 47.2%)	47.4% (42.7, 52.0%)	52.9% (46.0, 59.8%)	53.3% (44.2, 62.4%)
High	45.3% (43.0, 47.7%)	46.7% (44.3, 49.1%)	37.1% (32.9, 41.3%)	31.9% (25.3, 38.6%)	31.1% (22.7, 39.6%)
Personal income					
Low	52.5% (49.8, 55.2%)	51.6% (48.8, 54.4%)	58.7% (54.6, 62.8%)	57.1% (50.6, 63.5%)	58.3% (49.8, 66.8%)
Middle	24.9% (21.9, 27.9%)	25.1% (22.1, 28.1%)	22.7% (17.7, 27.8%)	24.3% (16.0, 32.5%)	21.4% (11.0, 31.8%)
High	22.6% (19.5, 25.6%)	23.3% (20.2, 26.4%)	18.5% (13.1, 23.9%)	18.7% (10.3, 27.0%)	20.3% (9.6, 31.0%)
AUDIT-C sum score ^a^					
Mean	4.5 (4.4, 4.5)	4.5 (4.4, 4.5)	5.2 (5.2, 5.3)	5.5 (5.4, 5.6)	5.7 (5.6, 5.8)
Score = 8 or more	17.8% (14.2, 21.4%)	17.8% (14.2, 21.4%)	25.8% (19.9, 31.6%)	24.2% (15.4, 33.1%)	28.5% (17.4, 39.5%)
Restrictions in day-to-day life					
not at all	4.6% (0.4, 8.8%)	3.9% (0.0, 8.2%)	8.0% (1.1, 14.9%)	11.3% (0.6, 22.1%)	18.1% (4.8, 31.4%)
to some degree	25.6% (22.1, 29.0%)	25.5% (21.9, 29.0%)	24.2% (18.5, 30.0%)	18.5% (9.2, 27.7%)	15.2% (3.9, 26.4%)
to a substantial degree	33.1% (30.1, 36.0%)	33.5% (30.5, 36.5%)	32.0% (27.1, 36.9%)	31.1% (23.4, 38.8%)	32.1% (21.6, 42.7%)
to a very high degree	36.8% (34.2, 39.3%)	37.1% (34.5, 39.7%)	35.8% (31.5, 40.1%)	39.1% (32.4, 45.8%)	34.6% (25.9, 43.3%)
Financial loss					
not at all	38.3% (35.6, 41.0%)	37.9% (35.1, 40.7%)	35.8% (31.1, 40.5%)	38.5% (31.1, 45.9%)	41.6% (32.0, 51.2%)
to some degree	34.8% (31.9, 37.8%)	35.3% (32.3, 38.3%)	32.3% (27.4, 37.3%)	28.3% (20.5, 36.0%)	21.4% (11.8, 31.1%)
to a substantial degree	15.3% (11.7, 18.8%)	15.3% (11.7, 18.9%)	17.4% (11.6, 23.2%)	16.7% (7.4, 26.0%)	20.0% (7.4, 32.5%)
to a very high degree	11.6% (8.2, 15.0%)	11.5% (8.0, 15.0%)	14.5% (8.9, 20.1%)	16.5% (7.7, 25.3%)	17.0% (5.9, 28.1%)
Direct SARS-CoV-2 contact	11.6% (8.1, 15.1%)	11.6% (8.0, 15.1%)	11.1% (5.3, 16.9%)	11.7% (2.8, 20.6%)	9.9% (0.0, 20.9%)

Note. 95% CI = 95% confidence intervals

^a^ Calculated for those who report any alcohol use: tobacco users, *n* = 9060; cannabis users, *n* = 3141; other illegal drug users, *n* = 1873

### Overall changes in use of alcohol, tobacco, cannabis and other illicit substances

**Fig. 1:**
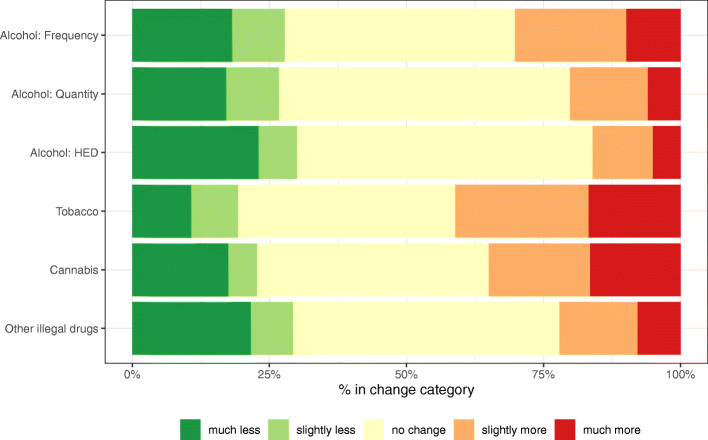
Proportion of respondents reporting any or no change in their substance use in the past month, calculated for alcohol users (*n* = 35,753), tobacco users (*n* = 9816), cannabis users (*n* = 3289), and other illegal drug users (*n* = 1961). HED = Heavy episodic drinking. Point estimates and confidence intervals for all change categories are reported in Appendix Table 2

In Fig. 1, we report changes in substance use in the first months of the pandemic for the three alcohol drinking indicators and the three other substances across the entire European sample. The most common response was no change in substance use, ranging from 40% for tobacco to 57% for heavy episodic drinking (proportion of respondents indicating no change in the remaining categories: frequency of drinking = 42%; quantity on drink days = 53%; cannabis = 42%; and other illicit substances = 49%).

A clear pattern emerges when contrasting increases with decreases in substance use. For two out of the three indicators of alcohol use (quantity and HED) the proportion of users reporting decreases in their use was greater than the proportion of users reporting increases in their use (any increase vs. any decrease in quantity: 20% vs. 26%; HED: 16% vs. 26%) as well as for other illegal substance use (18% vs. 23%). For frequency of alcohol use, a slightly higher proportion of users reported to have increased their use (30% vs. 27%), while for tobacco (39% vs. 18%) and cannabis users (30% vs. 19%), an increase in use was more pronounced.

This pattern was even more pronounced when comparing the proportion of users reporting “much more” to “much less” use; i.e., the responses at either extreme of the item categories. Only between 5 and 10% of respondents reported to have used much *more* alcohol on any of the three indicators, as opposed to between 17 to 20% who reported to have used *less* alcohol. A similar pattern was found for other illicit drug use (much more: 6% vs. much less: 17%), while for tobacco users this pattern was reversed (much more: 16% vs. much less: 10%). No apparent differences in the proportion of much more/less use were reported amongst cannabis users (much more: 14% vs. much less: 15%). The exact distribution for each substance use change indicator can be found in Appendix Table 2.

These descriptive findings are underpinned by the change indicator (see Appendix Table 2). On average, the quantity of alcohol consumed on drink days and the frequency of HED decreased significantly by − 6.6% (95% confidence interval [CI]: − 11.1, − 2.2%) and − 14.0% (95% CI: − 18.7, − 9.4%), respectively, while the frequency of drinking did not change substantially (2.3, 95% CI: − 1.9, 6.7%). Regarding increases in substance use, both tobacco (21.9, 95% CI: 14.7, 29.0%) and cannabis use (12.2, 95% CI: 0.4, 24.1%) increased significantly in the study population. The use of other illicit substances remained unchanged on average (− 7.2, 95% CI: − 24.3, 9.5%).

### Changes in use of alcohol, tobacco, cannabis and other illicit substances by country

**Fig. 2:**
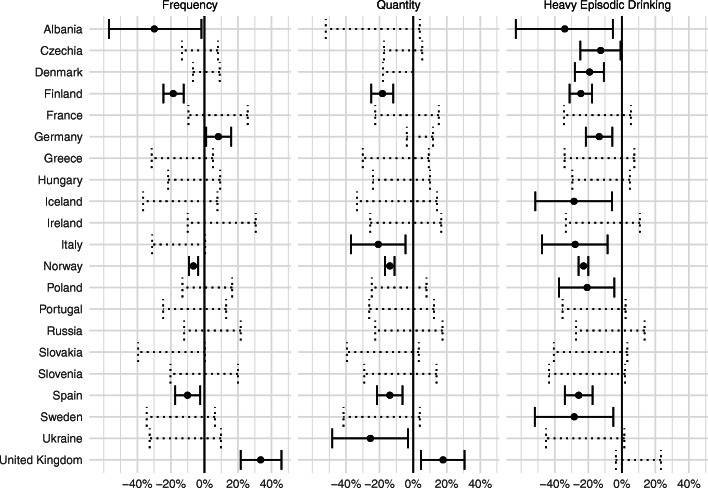
Indicator of change for three alcohol use indicators, calculated as the proportion of respondents reporting increases minus the proportion reporting decreases in their use, by country. Positive values (right hand side of vertical line) indicate increased use. Dashed lines indicate 95% confidence interval to overlap with 0, solid lines indicate non-overlapping, i.e. significant results

**Fig. 3:**
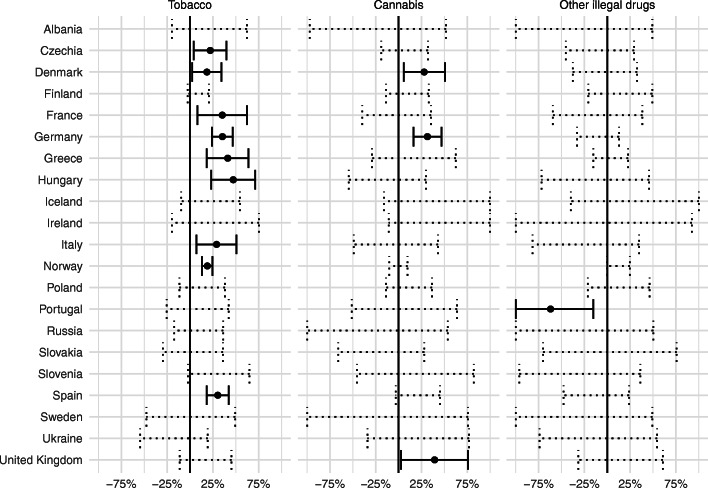
Indicator of change for tobacco, cannabis and other illegal drug use, calculated as the proportion of respondents reporting increases minus the proportion reporting decreases in their use, by country. Positive values (right hand side of vertical line) indicate increased use. Dashed lines indicate 95% confidence interval to overlap with 0, solid lines indicate non-overlapping, i.e., significant results

In Figs. 2 and 3, the reported changes are illustrated for each substance and by country. These figures corroborate the pattern observed for the entire European sample for both alcohol and tobacco use (compare with Fig. 1). Between 40 and 60% of respondents in seven countries (Czechia, Finland, France, Germany, Russia, Slovakia, Albania) reported no change in all three alcohol use indicators (frequency, quantity and HED). Among those who reported changes in their use, a greater proportion reported decreases rather than increases, in particular with regards to quantity and HED. For tobacco, however, a greater proportion of users reported to have changed their use and most indicated to have increased their use, rather than to have decreased their use.

For cannabis and other illicit drug use, patterns of changes were less clear (see Fig. 3). Taking into account 95% CIs, the crude indicator suggested an average increase in cannabis use in three countries only (Denmark, Germany, United Kingdom). For other illegal drug use, a significant average change was only observed in Portugal, where the crude indicator suggested a decreased use. The exact distribution of each substance-use change indicator by country can be found in Appendix Table 3.

## Discussion

Preliminary findings from a survey of more than 36,000 adult substance users in Europe suggest that use of alcohol, tobacco, cannabis and other illicit substances changed for about half of respondents during the first wave of the SARS-CoV-2 pandemic in spring 2020. Overall patterns suggest that more users tended to reduce rather than increase their alcohol use during this period, whilst the opposite was observed for tobacco and cannabis use. Taking into account uncertainty intervals, this overall pattern of change was only corroborated in some countries. For illicit drug use, there was no clear pattern of change in the overall sample.

Before discussing the implications of the results, we would like to highlight some key limitations of this study. First and foremost, we present self-reported changes in substance use, which were assessed retrospectively by survey respondents. Retrospective assessment of substance use is known to be affected by recall and social desirability biases, which lead to underreporting of use (for alcohol, see e.g. [34]; for illicit substance use, see e.g. [35]). The same biases, in addition to subjective interpretation, may have further distorted the meaning of the categories “much less” and “much more”, which may not indicate equal amounts/frequencies and likely depend on levels of baseline use. Further, we cannot exclude differences in reporting accuracy between substances, as potential stigmatization of illicit drug use is usually greater compared to legally regulated substances, resulting in a more pronounced social desirability bias for illicit drug use.

Second, we collected data from a convenience sample employing different dissemination strategies [25, 36], which may limit comparability across countries. Thus, results of our survey may not represent the population of substance users in either Europe as a whole, or with regard to the individual countries studied. As with other web-based surveys, certain subgroups are not well captured (e.g., older adults) or are potentially excluded from participation (e.g., those without internet access) altogether. Whilst this is a recognised problem in alcohol and other substance use surveys (for a discussion, see [37, 38]), we still see value in these data, especially in times of an acute public health crisis. Further, despite the relatively heterogeneous recruitment techniques used to engage substance users in different countries (for a summary, see [25]), the fact that we found consistent patterns across countries reduces the likelihood that our findings result from selection bias. Additionally, sample weights were applied to adjust for sample bias with respect to skewed representation of sex, age and education. In light of these limitations, we suggest the data presented here should be interpreted as trends for a general internet population that warrant further investigation at the country level. Future research in this area should employ a multi-faceted approach, combining routine statistics (such as sales or treatment demand data) as well as quantitative and qualitative surveys, to provide a more comprehensive and representative picture of substance use during the pandemic.

Keeping these limitations in mind, our findings reveal some important insights on substance use during the pandemic. For alcohol, a larger proportion of survey participants reported decreased use than increased use. This average decrease was particularly pronounced in relation to the frequency of HED events, where a decrease was reported twice as often as an increase. This European finding is consistent with findings from some (e.g., [13, 23]), albeit not all (e.g., [11, 39]), previous surveys of alcohol consumption during the pandemic. It remains unclear whether the changes presented here are phenomena specific to the current pandemic or merely represent normal year-to-year or seasonal fluctuations. However, as some countries have partially banned alcohol sales during the first months of the pandemic, future evaluation might provide more clarity on this matter [40]. Moreover, the different lockdown measures in response to the pandemic, including their duration and the respective impact on alcohol outlets such as bars and restaurants, add to the observed complexity of comparison across countries. Additional country analyses of the same data have indicated that the overall decreases in alcohol use found across Europe mask increases in consumption reported by previously heavy drinking individuals [41]. Routine clinical data from addiction outpatient services in Barcelona, Spain, suggest a doubling of positive alcohol urine screening tests [42], while alcohol withdrawal treatment demand in Bangalore, India, has reportedly spiked following lockdowns which included a complete halt of alcohol sales [43]. Potentially increasing alcohol use among heavier drinkers can be linked to an elevated risk of complications following a SARS-CoV-2 infection [44], but also to other alcohol-attributable harm, thus, an average decrease of alcohol use does not necessarily imply a lower alcohol-attributable societal burden.

For tobacco use, about twice as many survey participants who are current smokers reported to increase their use compared to those reporting a decrease, which also reflects findings from several other surveys (e.g. [11, 39]) and trend studies [45]. This increase in smoking could be seen as a reaction to the stress in experiencing the pandemic [46, 47] or to more time spent at home, where less restrictive smoking policies exist than at the workplace. Our results are indicative of an overall increase in tobacco use which is also corroborated by preliminary sales data available from Germany [48], Norway [49], and the UK [50]. While possible changes in unrecorded tobacco supply are yet to be considered to determine changes in population level tobacco use, an upward trend would constitute a severe setback in reaching global goals to reduce smoking prevalence [51] and the attributable burden, both of which remain high in the European region [52, 53]. Given that one in six EU deaths from non-communicable diseases was attributable to tobacco use in 2017 [54], this could hamper the projected achievements of reductions in mortality from cancers and cardiovascular diseases in the region (for a recent update on progress in reducing the non-communicable disease burden, see [55]).

For cannabis, an overall of current users to increase their consumption was identified, although country-level analyses only corroborated this pattern for 3 out of 21 countries. This pattern is consistent with longitudinal findings from the Netherlands [56] and cross-sectional survey results from Germany [57]. Further, the results may, to some extent, represent a continuation of trajectories observed in recent years [58, 59]. The increased use may be related both to stress as well as the arrival of unexpected leisure time or boredom related to lockdown. It has been noted that during the early months of the pandemic, darknet sales of cannabis in Europe increased and shifted to smaller quantities [20]. While these data provide information on changes in purchasing behaviour during this period, they should be triangulated with survey data in order to corroborate changes in use behaviour. However, unlike alcohol and tobacco use (see e.g., European Health Interview Survey [60]), there is no ongoing comparative general population survey assessing cannabis use in Europe. In light of increasing potency levels [61], treatment demand [58], unintentional intoxications (e.g. among infants [62]), and possibly increasing use during the pandemic, comparative cannabis use survey data is warranted.

For other illicit substances, the sample size of users was relatively small so we did not observe a clear pattern of change in the overall sample and, at country level, an average decrease in Portugal only. Consequently, our findings reveal no consistent pattern across countries, which is not unexpected given that we asked about a heterogeneous class of substances (“illicit drugs (other than cannabis)”) and changes in use behavior will largely depend on the type of substance [24]. For instance, use of substances such as 3,4-Methylenedioxy​methamphetamine (MDMA) may decrease with the closure of the night-time economy, while amphetamine use may increase if used to enhance productivity and to cope with stress [63]. Notably, increases in opioid overdose emergency admissions and deaths following lockdown have been reported in some US states [64–66], while German data from the first 6 months of 2020 indicate a 13% increase of drug overdose deaths as compared to 2019 [67], which may be due in part to changing purity and resulting uncertainties for titration [18].

Finally, we would like to highlight that, on average, users reported increases in their tobacco and cannabis use but decreases in their alcohol use during the first wave of the pandemic. One possible explanation may be related to the different contexts of use of these substances. While most tobacco and cannabis users may use the drug(s) regardless of social gatherings, alcohol use remains a social drug for most users. Another hypothesis is that users of tobacco or cannabis may have experienced pandemic-induced stress to a larger extent or more intensely than alcohol users, and that they attempted to cope with such stress by increasing their substance use. However, we cannot rule out alternative explanations, such as differential sampling bias (heavy users of certain substances, who are also more likely to increase their use, are more prone to use the internet than other substance users) or differences in use patterns prior to the pandemic. Future studies examining changes of substance use should therefore not only account for pre-pandemic use patterns, but also for context of use (e.g., social, party, work).

## Conclusions

Overall, the findings of our survey indicate that substance use behaviour changed for about every other user during the first months of the pandemic in Europe. Substantially more respondents reported decreases rather than increases in their alcohol use, while the opposite pattern could be observed for tobacco and cannabis use. The changes in substance use in Europe reported here could result from a number of factors, including availability, changing social contexts and stress, while being moderated by differing characteristics of substance use, consumption contexts and different consumption cultures of the countries and substances studied. The pandemic is an ongoing natural experiment that offers a multitude of opportunities to study these mechanisms, thus allowing for improvements in our understanding of the societal roles of substance use, its susceptibility to change, and strengthening policy measures to reduce harm.

## Supplementary Information

**Additional file 1: Appendix Table 1.** Unweighted sample characteristics by country. **Appendix Table 2.** Reported changes for each substance for the entire survey sample. **Appendix Table 3.** Reported changes for each substance by country.

## Data Availability

The datasets generated during and/or analysed during the current study are available in the figshare repository, 10.6084/m9.figshare.14315606.v1 [33].

## Abbreviations

SARS-CoV-2: Severe acute respiratory syndrome coronavirus 2
HED: Heavy episodic drinking
AUDIT-C: Alcohol Use Disorder Identification Test – Consumption section
