# Supplementary material for: Use of alcohol, tobacco, cannabis, and other substances during the first wave of the SARS-CoV-2 pandemic in Europe: a survey on 36,000 European substance users
Source: Subst Abuse Treat Prev Policy. 2021 Apr 26;16:36. doi: 10.1186/s13011-021-00373-y (PMC8072737; doi:10.1186/s13011-021-00373-y)
Supplement: Supplementary file 1 — Additional file 1: Appendix Table 1. Unweighted sample characteristics by country. Appendix Table 2. Reported changes for each substance for the entire survey sample. Appendix Table 3. Reported changes for each substance by country. [file 13011_2021_373_MOESM1_ESM.docx]

# Appendix

For manuscript: “Use of alcohol, tobacco, cannabis, and other substances during the first wave of the SARS-CoV-2 pandemic in Europe: A survey on 36,000 European substance users”

Authors: Jakob Manthey, Carolin Kilian, Sinclair Carr, Miroslav Bartak, Kim Bloomfield, Fleur Braddick, Antoni Gual, Maria Neufeld, Amy O’Donnell, Benjamin Petruzelka, Vladimir Rogalewicz, Ingeborg Rossow, Bernd Schulte, Jürgen Rehm

| **Appendix Table 1.** Unweighted sample characteristics by country | | | | | | |
| --- | --- | --- | --- | --- | --- | --- |
|  | | Any substance users | Alcohol users | Tobacco users | Cannabis users | Other illegal drug users |
| Country |  | % (95% CI) or n | | | | |
| Albania | n | 221 | 204 | 67 | 22 | 17 |
|  | Sex |  |  |  |  |  |
|  | Female | 68.8% (62.6%, 74.9%) | 69.6% (63.2%, 76.0%) | 50.7% (38.5%, 63.0%) | 50.0% (27.3%, 72.7%) | 58.8% (32.7%, 84.9%) |
|  | Male | 29.9% (23.8%, 35.9%) | 28.9% (22.6%, 35.2%) | 44.8% (32.6%, 57.0%) | 40.9% (18.6%, 63.2%) | 35.3% (10.0%, 60.6%) |
|  | Other | 1.4% (0.0%, 2.9%) | 1.5% (0.0%, 3.1%) | 4.5% (0.0%, 9.6%) | 9.1% (0.0%, 22.1%) | 5.9% (0.0%, 18.4%) |
|  | Age group |  |  |  |  |  |
|  | 18-34 | 71.9% (66.0%, 77.9%) | 74.0% (68.0%, 80.1%) | 61.2% (49.2%, 73.2%) | 72.7% (52.5%, 92.9%) | 70.6% (46.4%, 94.7%) |
|  | 35-54 | 19.5% (14.2%, 24.7%) | 17.6% (12.4%, 22.9%) | 23.9% (13.4%, 34.4%) | 18.2% (0.7%, 35.7%) | 23.5% (1.0%, 46.0%) |
|  | 55+ | 8.6% (4.9%, 12.3%) | 8.3% (4.5%, 12.2%) | 14.9% (6.2%, 23.7%) | 9.1% (0.0%, 22.1%) | 5.9% (0.0%, 18.4%) |
|  | Education |  |  |  |  |  |
|  | Low | 2.3% (0.3%, 4.2%) | 2.0% (0.0%, 3.9%) | 4.5% (0.0%, 9.6%) | 4.5% (0.0%, 14.0%) | 5.9% (0.0%, 18.4%) |
|  | Middle | 45.7% (39.1%, 52.3%) | 45.6% (38.7%, 52.5%) | 41.8% (29.7%, 53.9%) | 31.8% (10.7%, 53.0%) | 41.2% (15.1%, 67.3%) |
|  | High | 52.0% (45.4%, 58.7%) | 52.5% (45.5%, 59.4%) | 53.7% (41.5%, 66.0%) | 63.6% (41.8%, 85.5%) | 52.9% (26.5%, 79.4%) |
| Czechia | n | 1,467 | 1,434 | 456 | 197 | 101 |
|  | Sex |  |  |  |  |  |
|  | Female | 69.2% (66.8%, 71.6%) | 69.4% (67.0%, 71.8%) | 68.0% (63.7%, 72.3%) | 59.4% (52.5%, 66.3%) | 52.5% (42.6%, 62.4%) |
|  | Male | 30.6% (28.2%, 33.0%) | 30.4% (28.0%, 32.8%) | 31.6% (27.3%, 35.9%) | 39.6% (32.7%, 46.5%) | 45.5% (35.7%, 55.4%) |
|  | Other | 0.2% (0.0%, 0.4%) | 0.2% (0.0%, 0.4%) | 0.4% (0.0%, 1.0%) | 1.0% (0.0%, 2.4%) | 2.0% (0.0%, 4.7%) |
|  | Age group |  |  |  |  |  |
|  | 18-34 | 46.3% (43.7%, 48.8%) | 46.7% (44.1%, 49.3%) | 47.6% (43.0%, 52.2%) | 61.4% (54.6%, 68.3%) | 60.4% (50.7%, 70.1%) |
|  | 35-54 | 42.5% (39.9%, 45.0%) | 42.3% (39.7%, 44.8%) | 42.3% (37.8%, 46.9%) | 33.0% (26.4%, 39.6%) | 32.7% (23.4%, 42.0%) |
|  | 55+ | 11.2% (9.6%, 12.9%) | 11.0% (9.4%, 12.6%) | 10.1% (7.3%, 12.9%) | 5.6% (2.3%, 8.8%) | 6.9% (1.9%, 12.0%) |
|  | Education |  |  |  |  |  |
|  | Low | 2.1% (1.4%, 2.8%) | 1.9% (1.2%, 2.6%) | 4.2% (2.3%, 6.0%) | 2.5% (0.3%, 4.8%) | 1.0% (0.0%, 3.0%) |
|  | Middle | 32.7% (30.3%, 35.1%) | 32.1% (29.7%, 34.5%) | 41.9% (37.3%, 46.4%) | 37.6% (30.7%, 44.4%) | 34.7% (25.2%, 44.1%) |
|  | High | 65.2% (62.7%, 67.6%) | 66.0% (63.6%, 68.5%) | 53.9% (49.4%, 58.5%) | 59.9% (53.0%, 66.8%) | 64.4% (54.9%, 73.9%) |
| Denmark | n | 2,473 | 2,437 | 710 | 247 | 113 |
|  | Sex |  |  |  |  |  |
|  | Female | 78.2% (76.6%, 79.8%) | 78.2% (76.6%, 79.9%) | 76.9% (73.8%, 80.0%) | 64.4% (58.4%, 70.4%) | 60.2% (51.0%, 69.3%) |
|  | Male | 21.4% (19.8%, 23.0%) | 21.4% (19.7%, 23.0%) | 22.8% (19.7%, 25.9%) | 34.8% (28.8%, 40.8%) | 39.8% (30.7%, 49.0%) |
|  | Other | 0.4% (0.2%, 0.7%) | 0.4% (0.2%, 0.7%) | 0.3% (0.0%, 0.7%) | 0.8% (0.0%, 1.9%) | - |
|  | Age group |  |  |  |  |  |
|  | 18-34 | 64.8% (62.9%, 66.7%) | 65.0% (63.1%, 66.9%) | 70.1% (66.8%, 73.5%) | 72.9% (67.3%, 78.5%) | 69.9% (61.3%, 78.5%) |
|  | 35-54 | 26.0% (24.2%, 27.7%) | 25.9% (24.1%, 27.6%) | 24.1% (20.9%, 27.2%) | 20.6% (15.6%, 25.7%) | 23.0% (15.1%, 30.9%) |
|  | 55+ | 9.2% (8.1%, 10.4%) | 9.2% (8.0%, 10.3%) | 5.8% (4.1%, 7.5%) | 6.5% (3.4%, 9.6%) | 7.1% (2.3%, 11.9%) |
|  | Education |  |  |  |  |  |
|  | Low | 5.5% (4.6%, 6.4%) | 5.4% (4.5%, 6.3%) | 7.9% (5.9%, 9.9%) | 13.4% (9.1%, 17.6%) | 10.6% (4.9%, 16.4%) |
|  | Middle | 34.8% (32.9%, 36.7%) | 35.0% (33.1%, 36.9%) | 37.7% (34.2%, 41.3%) | 40.9% (34.7%, 47.1%) | 33.6% (24.8%, 42.5%) |
|  | High | 59.7% (57.7%, 61.6%) | 59.6% (57.6%, 61.5%) | 54.4% (50.7%, 58.0%) | 45.7% (39.5%, 52.0%) | 55.8% (46.5%, 65.1%) |
| Finland | n | 3,540 | 3,471 | 966 | 227 | 123 |
|  | Sex |  |  |  |  |  |
|  | Female | 76.3% (74.9%, 77.7%) | 76.4% (75.0%, 77.8%) | 73.9% (71.1%, 76.7%) | 46.7% (40.2%, 53.2%) | 47.2% (38.2%, 56.1%) |
|  | Male | 22.7% (21.4%, 24.1%) | 22.6% (21.2%, 24.0%) | 24.9% (22.2%, 27.7%) | 50.7% (44.1%, 57.2%) | 48.0% (39.0%, 56.9%) |
|  | Other | 1.0% (0.7%, 1.3%) | 1.0% (0.7%, 1.3%) | 1.1% (0.5%, 1.8%) | 2.6% (0.5%, 4.7%) | 4.9% (1.0%, 8.7%) |
|  | Age group |  |  |  |  |  |
|  | 18-34 | 11.0% (10.0%, 12.1%) | 11.2% (10.2%, 12.3%) | 14.1% (11.9%, 16.3%) | 25.1% (19.4%, 30.8%) | 25.2% (17.4%, 33.0%) |
|  | 35-54 | 67.1% (65.5%, 68.6%) | 67.1% (65.5%, 68.6%) | 67.1% (64.1%, 70.0%) | 61.7% (55.3%, 68.0%) | 63.4% (54.8%, 72.0%) |
|  | 55+ | 21.9% (20.5%, 23.3%) | 21.7% (20.3%, 23.1%) | 18.8% (16.4%, 21.3%) | 13.2% (8.8%, 17.7%) | 11.4% (5.7%, 17.1%) |
|  | Education |  |  |  |  |  |
|  | Low | 3.1% (2.5%, 3.6%) | 2.9% (2.4%, 3.5%) | 6.0% (4.5%, 7.5%) | 9.3% (5.5%, 13.0%) | 9.8% (4.4%, 15.1%) |
|  | Middle | 38.9% (37.3%, 40.5%) | 38.5% (36.9%, 40.1%) | 52.3% (49.1%, 55.4%) | 48.9% (42.3%, 55.5%) | 47.2% (38.2%, 56.1%) |
|  | High | 58.0% (56.4%, 59.6%) | 58.6% (57.0%, 60.2%) | 41.7% (38.6%, 44.8%) | 41.9% (35.4%, 48.3%) | 43.1% (34.2%, 52.0%) |
| France | n | 337 | 314 | 137 | 65 | 40 |
|  | Sex |  |  |  |  |  |
|  | Female | 61.7% (56.5%, 66.9%) | 63.4% (58.0%, 68.7%) | 54.7% (46.3%, 63.2%) | 43.1% (30.7%, 55.4%) | 35.0% (19.6%, 50.4%) |
|  | Male | 37.7% (32.5%, 42.9%) | 36.0% (30.6%, 41.3%) | 43.8% (35.4%, 52.2%) | 53.8% (41.4%, 66.3%) | 60.0% (44.1%, 75.9%) |
|  | Other | 0.6% (0.0%, 1.4%) | 0.6% (0.0%, 1.5%) | 1.5% (0.0%, 3.5%) | 3.1% (0.0%, 7.4%) | 5.0% (0.0%, 12.1%) |
|  | Age group |  |  |  |  |  |
|  | 18-34 | 22.0% (17.5%, 26.4%) | 22.9% (18.3%, 27.6%) | 26.3% (18.8%, 33.7%) | 35.4% (23.4%, 47.3%) | 30.0% (15.2%, 44.8%) |
|  | 35-54 | 36.8% (31.6%, 42.0%) | 37.6% (32.2%, 43.0%) | 37.2% (29.0%, 45.4%) | 36.9% (24.9%, 49.0%) | 37.5% (21.8%, 53.2%) |
|  | 55+ | 41.2% (36.0%, 46.5%) | 39.5% (34.1%, 44.9%) | 36.5% (28.3%, 44.7%) | 27.7% (16.5%, 38.9%) | 32.5% (17.3%, 47.7%) |
|  | Education |  |  |  |  |  |
|  | Low | 3.6% (1.6%, 5.5%) | 2.5% (0.8%, 4.3%) | 5.1% (1.4%, 8.8%) | 6.2% (0.2%, 12.2%) | 10.0% (0.3%, 19.7%) |
|  | Middle | 12.5% (8.9%, 16.0%) | 11.1% (7.6%, 14.6%) | 16.1% (9.8%, 22.3%) | 20.0% (10.0%, 30.0%) | 27.5% (13.0%, 42.0%) |
|  | High | 84.0% (80.0%, 87.9%) | 86.3% (82.5%, 90.1%) | 78.8% (71.9%, 85.8%) | 73.8% (62.9%, 84.8%) | 62.5% (46.8%, 78.2%) |
| Germany | n | 1,565 | 1,517 | 588 | 333 | 188 |
|  | Sex |  |  |  |  |  |
|  | Female | 50.9% (48.4%, 53.3%) | 51.4% (48.8%, 53.9%) | 42.9% (38.8%, 46.9%) | 35.1% (30.0%, 40.3%) | 39.4% (32.3%, 46.4%) |
|  | Male | 48.2% (45.7%, 50.7%) | 47.7% (45.1%, 50.2%) | 56.0% (51.9%, 60.0%) | 62.5% (57.2%, 67.7%) | 59.0% (51.9%, 66.1%) |
|  | Other | 1.0% (0.5%, 1.4%) | 1.0% (0.5%, 1.5%) | 1.2% (0.3%, 2.1%) | 2.4% (0.7%, 4.1%) | 1.6% (0.0%, 3.4%) |
|  | Age group |  |  |  |  |  |
|  | 18-34 | 36.9% (34.5%, 39.3%) | 37.8% (35.4%, 40.3%) | 38.8% (34.8%, 42.7%) | 54.4% (49.0%, 59.7%) | 56.9% (49.8%, 64.1%) |
|  | 35-54 | 42.3% (39.8%, 44.8%) | 42.3% (39.8%, 44.8%) | 41.0% (37.0%, 45.0%) | 37.5% (32.3%, 42.8%) | 34.6% (27.7%, 41.4%) |
|  | 55+ | 20.8% (18.8%, 22.8%) | 19.8% (17.8%, 21.9%) | 20.2% (17.0%, 23.5%) | 8.1% (5.2%, 11.1%) | 8.5% (4.5%, 12.5%) |
|  | Education |  |  |  |  |  |
|  | Low | 14.5% (12.8%, 16.3%) | 13.8% (12.1%, 15.6%) | 19.6% (16.3%, 22.8%) | 12.6% (9.0%, 16.2%) | 16.0% (10.7%, 21.2%) |
|  | Middle | 28.0% (25.8%, 30.2%) | 27.8% (25.5%, 30.0%) | 33.7% (29.8%, 37.5%) | 35.4% (30.3%, 40.6%) | 30.9% (24.2%, 37.5%) |
|  | High | 57.5% (55.1%, 60.0%) | 58.4% (55.9%, 60.9%) | 46.8% (42.7%, 50.8%) | 52.0% (46.6%, 57.3%) | 53.2% (46.0%, 60.4%) |
| Greece | n | 536 | 531 | 248 | 84 | 38 |
|  | Sex |  |  |  |  |  |
|  | Female | 59.3% (55.2%, 63.5%) | 59.1% (54.9%, 63.3%) | 59.7% (53.5%, 65.8%) | 50.0% (39.1%, 60.9%) | 42.1% (25.7%, 58.6%) |
|  | Male | 40.3% (36.1%, 44.5%) | 40.5% (36.3%, 44.7%) | 39.9% (33.8%, 46.1%) | 48.8% (37.9%, 59.7%) | 57.9% (41.4%, 74.3%) |
|  | Other | 0.4% (0.0%, 0.9%) | 0.4% (0.0%, 0.9%) | 0.4% (0.0%, 1.2%) | 1.2% (0.0%, 3.6%) | - |
|  | Age group |  |  |  |  |  |
|  | 18-34 | 52.8% (48.6%, 57.0%) | 53.1% (48.8%, 57.4%) | 48.8% (42.5%, 55.1%) | 66.7% (56.4%, 77.0%) | 57.9% (41.4%, 74.3%) |
|  | 35-54 | 40.7% (36.5%, 44.8%) | 40.3% (36.1%, 44.5%) | 44.8% (38.5%, 51.0%) | 27.4% (17.6%, 37.1%) | 34.2% (18.4%, 50.0%) |
|  | 55+ | 6.5% (4.4%, 8.6%) | 6.6% (4.5%, 8.7%) | 6.5% (3.4%, 9.5%) | 6.0% (0.8%, 11.1%) | 7.9% (0.0%, 16.9%) |
|  | Education |  |  |  |  |  |
|  | Low | 0.6% (0.0%, 1.2%) | 0.4% (0.0%, 0.9%) | 0.8% (0.0%, 1.9%) | 1.2% (0.0%, 3.6%) | - |
|  | Middle | 23.7% (20.1%, 27.3%) | 23.9% (20.3%, 27.6%) | 21.8% (16.6%, 26.9%) | 20.2% (11.5%, 29.0%) | 10.5% (0.3%, 20.7%) |
|  | High | 75.7% (72.1%, 79.4%) | 75.7% (72.0%, 79.4%) | 77.4% (72.2%, 82.7%) | 78.6% (69.6%, 87.5%) | 89.5% (79.3%, 99.7%) |
| Hungary | n | 456 | 434 | 189 | 68 | 39 |
|  | Sex |  |  |  |  |  |
|  | Female | 64.5% (60.1%, 68.9%) | 63.8% (59.3%, 68.4%) | 57.1% (50.0%, 64.3%) | 51.5% (39.3%, 63.7%) | 51.3% (34.9%, 67.7%) |
|  | Male | 35.3% (30.9%, 39.7%) | 35.9% (31.4%, 40.5%) | 42.3% (35.2%, 49.4%) | 47.1% (34.9%, 59.2%) | 46.2% (29.8%, 62.5%) |
|  | Other | 0.2% (0.0%, 0.7%) | 0.2% (0.0%, 0.7%) | 0.5% (0.0%, 1.6%) | 1.5% (0.0%, 4.4%) | 2.6% (0.0%, 7.8%) |
|  | Age group |  |  |  |  |  |
|  | 18-34 | 40.8% (36.3%, 45.3%) | 42.6% (38.0%, 47.3%) | 43.4% (36.3%, 50.5%) | 63.2% (51.5%, 75.0%) | 51.3% (34.9%, 67.7%) |
|  | 35-54 | 36.8% (32.4%, 41.3%) | 36.6% (32.1%, 41.2%) | 34.9% (28.1%, 41.8%) | 25.0% (14.4%, 35.6%) | 41.0% (24.9%, 57.2%) |
|  | 55+ | 22.4% (18.5%, 26.2%) | 20.7% (16.9%, 24.6%) | 21.7% (15.8%, 27.6%) | 11.8% (3.9%, 19.6%) | 7.7% (0.0%, 16.4%) |
|  | Education |  |  |  |  |  |
|  | Low | 18.9% (15.3%, 22.5%) | 18.4% (14.8%, 22.1%) | 32.3% (25.5%, 39.0%) | 23.5% (13.2%, 33.9%) | 12.8% (1.8%, 23.8%) |
|  | Middle | 25.9% (21.8%, 29.9%) | 26.0% (21.9%, 30.2%) | 22.8% (16.7%, 28.8%) | 26.5% (15.7%, 37.2%) | 33.3% (17.9%, 48.8%) |
|  | High | 55.3% (50.7%, 59.8%) | 55.5% (50.8%, 60.2%) | 45.0% (37.8%, 52.1%) | 50.0% (37.8%, 62.2%) | 53.8% (37.5%, 70.2%) |
| Iceland | n | 500 | 477 | 107 | 14 | 12 |
|  | Sex |  |  |  |  |  |
|  | Female | 81.6% (78.2%, 85.0%) | 81.6% (78.1%, 85.0%) | 74.8% (66.4%, 83.1%) | 71.4% (44.4%, 98.5%) | 66.7% (35.4%, 98.0%) |
|  | Male | 18.4% (15.0%, 21.8%) | 18.4% (15.0%, 21.9%) | 25.2% (16.9%, 33.6%) | 28.6% (1.5%, 55.6%) | 33.3% (2.0%, 64.6%) |
|  | Other | - | - | - | - | - |
|  | Age group |  |  |  |  |  |
|  | 18-34 | 9.2% (6.7%, 11.7%) | 9.4% (6.8%, 12.1%) | 12.1% (5.9%, 18.4%) | 50.0% (20.0%, 80.0%) | 25.0% (0.0%, 53.7%) |
|  | 35-54 | 45.0% (40.6%, 49.4%) | 45.5% (41.0%, 50.0%) | 48.6% (39.0%, 58.2%) | 35.7% (7.0%, 64.4%) | 25.0% (0.0%, 53.7%) |
|  | 55+ | 45.8% (41.4%, 50.2%) | 45.1% (40.6%, 49.6%) | 39.3% (29.8%, 48.7%) | 14.3% (0.0%, 35.3%) | 50.0% (16.8%, 83.2%) |
|  | Education |  |  |  |  |  |
|  | Low | 8.6% (6.1%, 11.1%) | 7.5% (5.2%, 9.9%) | 15.0% (8.1%, 21.8%) | 7.1% (0.0%, 22.6%) | 8.3% (0.0%, 26.7%) |
|  | Middle | 20.2% (16.7%, 23.7%) | 19.5% (15.9%, 23.1%) | 28.0% (19.4%, 36.7%) | 42.9% (13.2%, 72.5%) | 33.3% (2.0%, 64.6%) |
|  | High | 71.2% (67.2%, 75.2%) | 73.0% (69.0%, 77.0%) | 57.0% (47.5%, 66.5%) | 50.0% (20.0%, 80.0%) | 58.3% (25.6%, 91.1%) |
| Ireland | n | 493 | 483 | 92 | 30 | 17 |
|  | Sex |  |  |  |  |  |
|  | Female | 69.4% (65.3%, 73.5%) | 70.0% (65.9%, 74.1%) | 62.0% (51.8%, 72.1%) | 43.3% (24.5%, 62.2%) | 29.4% (5.3%, 53.6%) |
|  | Male | 30.0% (26.0%, 34.1%) | 29.4% (25.3%, 33.5%) | 38.0% (27.9%, 48.2%) | 56.7% (37.8%, 75.5%) | 70.6% (46.4%, 94.7%) |
|  | Other | 0.6% (0.0%, 1.3%) | 0.6% (0.0%, 1.3%) | - | - | - |
|  | Age group |  |  |  |  |  |
|  | 18-34 | 18.1% (14.6%,21.5%) | 18.4% (15.0%, 21.9%) | 22.8% (14.1%, 31.6%) | 40.0% (21.4%, 58.6%) | 35.3% (10.0%, 60.6%) |
|  | 35-54 | 56.4% (52.0%, 60.8%) | 55.7% (51.2%, 60.1%) | 58.7% (48.4%, 68.9%) | 53.3% (34.4%, 72.3%) | 58.8% (32.7%, 84.9%) |
|  | 55+ | 25.6% (21.7%, 29.4%) | 25.9% (22.0%, 29.8%) | 18.5% (10.4%, 26.6%) | 6.7% (0.0%, 16.1%) | 5.9% (0.0%, 18.4%) |
|  | Education |  |  |  |  |  |
|  | Low | 1.0% (0.1%, 1.9%) | 1.0% (0.1%, 1.9%) | 1.1% (0.0%, 3.2%) | 3.3% (0.0%, 10.2%) | 5.9% (0.0%, 18.4%) |
|  | Middle | 7.1% (4.8%, 9.4%) | 7.2% (4.9%, 9.6%) | 10.9% (4.4%, 17.4%) | 10.0% (0.0%, 21.4%) | 5.9% (0.0%, 18.4%) |
|  | High | 91.9% (89.5%, 94.3%) | 91.7% (89.3%, 94.2%) | 88.0% (81.3%, 94.8%) | 86.7% (73.8%, 99.6%) | 88.2% (71.2%, 100.0%) |
| Italy | n | 756 | 687 | 289 | 98 | 52 |
|  | Sex |  |  |  |  |  |
|  | Female | 63.6% (60.2%, 67.1%) | 65.4% (61.8%, 68.9%) | 58.8% (53.1%, 64.5%) | 50.0% (39.9%, 60.1%) | 46.2% (32.1%, 60.2%) |
|  | Male | 36.2% (32.8%, 39.7%) | 34.5% (30.9%, 38.1%) | 40.8% (35.q%, 46.5%) | 49.0% (38.9%, 59.1%) | 51.9% (37.9%, 66.0%) |
|  | Other | 0.1% (0.0%, 0.4%) | 0.1% (0.0%, 0.4%) | 0.3% (0.0%, 1.0%) | 1.0% (0.0%, 3.0%) | 1.9% (0.0%, 5.8%) |
|  | Age group |  |  |  |  |  |
|  | 18-34 | 19.6% (16.7%, 22.4%) | 21.0% (17.9%, 24.0%) | 19.7% (15.1%, 24.3%) | 25.5% (16.7%, 34.3%) | 15.4% (5.2%, 25.5%) |
|  | 35-54 | 41.8% (38.3%, 45.3%) | 43.1% (39.4%, 46.8%) | 41.9% (36.1%, 47.6%) | 44.9% (34.9%, 54.9%) | 42.3% (28.4%, 56.2%) |
|  | 55+ | 38.6% (35.2%, 42.1%) | 36.0% (32.4%, 39.6%) | 38.4% (32.8%, 44.0%) | 29.6% (20.4%, 38.8%) | 42.3% (28.4%, 56.2%) |
|  | Education |  |  |  |  |  |
|  | Low | 6.9% (5.1%, 8.7%) | 3.2% (1.9%, 4.5%) | 14.9% (10.8%, 19.0%) | 13.3% (6.4%, 20.1%) | 15.4% (5.2%, 25.5%) |
|  | Middle | 22.8% (19.8%, 25.7%) | 22.1% (19.0%, 25.2%) | 27.0% (21.8%, 32.1%) | 24.5% (15.8%, 33.2%) | 25.0% (12.8%, 37.2%) |
|  | High | 70.4% (67.1%, 73.6%) | 74.7% (71.4%, 77.9%) | 58.1% (52.4%, 63.9%) | 62.2% (52.5%, 72.0%) | 59.6% (45.8%, 73.4%) |
| Norway | n | 15,762 | 15,520 | 3,445 | 976 | 664 |
|  | Sex |  |  |  |  |  |
|  | Female | 72.5% (71.8%, 73.2%) | 72.4% (71.7%, 73.1%) | 68.8% (67.3%, 70.4%) | 49.4% (46.2%, 52.5%) | 47.1% (43.3%, 50.9%) |
|  | Male | 27.4% (26.7%, 28.1%) | 27.5% (26.8%, 28.2%) | 30.9% (29.3%, 32.4%) | 49.5% (46.3%, 52.6%) | 51.7% (47. 8%, 55.5%) |
|  | Other | 0.2% (0.1%, 0.2%) | 0.1% (0.1%, 0.2%) | 0.3% (0.1%, 0.5%) | 1.1% (0.5%, 1.8%) | 1.2% (0.4%, 2.0%) |
|  | Age group |  |  |  |  |  |
|  | 18-34 | 35.2% (34.5%, 36.0%) | 35.5% (34.7%, 36.2%) | 37.0% (35.3%, 38.6%) | 63.0% (60.0%, 66.0%) | 64.6% (61.0%, 68.3%) |
|  | 35-54 | 49.4% (48.7%, 50.2%) | 49.4% (48.6%, 50.2%) | 49.5% (47.8%, 51.2%) | 30.9% (28.0%, 33.8%) | 28.9% (25.5%, 32.4%) |
|  | 55+ | 15.3% (14.8%, 15.9%) | 15.1% (14.6%, 15.7%) | 13.6% (12.4%, 14.7%) | 6.0% (4.5%, 7.5%) | 6.5% (4.6%, 8.4%) |
|  | Education |  |  |  |  |  |
|  | Low | 3.7% (3.4%, 4.0%) | 3.6% (3.3%, 3.9%) | 7.1% (6.3%, 8.0%) | 6.8% (5.2%, 8.3%) | 6.6% (4.7%, 8.5%) |
|  | Middle | 29.7% (29.0%, 30.4%) | 29.3% (28.6%, 30.0%) | 41.1% (39.5%, 42.7%) | 43.2% (40.1%, 46.4%) | 48.2% (44.4%, 52.0%) |
|  | High | 66.7% (65.9%, 67.4%) | 67.1% (66.4%, 67.9%) | 51.8% (50.1%, 53.5%) | 50.0% (46.9%, 53.1%) | 45.2% (41.4%, 49.0%) |
| Poland | n | 1,104 | 1090 | 367 | 291 | 161 |
|  | Sex |  |  |  |  |  |
|  | Female | 37.1% (34.3%, 40.0%) | 36.9% (34.0%, 39.7%) | 38.4% (33.4%, 43.4%) | 25.4% (20.4%, 30.5%) | 31.7% (24.4%, 38.9%) |
|  | Male | 62.6% (59.7%, 65.4%) | 62.8% (60.0%, 65.7%) | 61.3% (56.3%, 66.3%) | 73.5% (68.4%, 78.6%) | 67.7% (60.4%, 75.0%) |
|  | Other | 0.3% (0.0%, 0.6%) | 0.3% (0.0%, 0.6%) | 0.3% (0.0%, 0.8%) | 1.0% (0.0%, 2.2%) | 0.6% (0.0%, 1.8%) |
|  | Age group |  |  |  |  |  |
|  | 18-34 | 62.6% (59.7%, 65.4%) | 62.9% (60.1%, 65.8%) | 68.1% (63.3%, 72.9%) | 83.2% (78.8%, 87.5%) | 82.0% (76.0%, 88.0%) |
|  | 35-54 | 31.9% (29.1%, 34.6%) | 31.7% (29.0%, 34.5%) | 28.1% (23.4%, 32.7%) | 16.5% (12.2%, 20.8%) | 18.0% (12.0%, 24.0%) |
|  | 55+ | 5.5% (4.2%, 6.9%) | 5.3% (4.0%, 6.7%) | 3.8% (1.8%, 5.8%) | 0.3% (0.0%, 1.0%) | - |
|  | Education |  |  |  |  |  |
|  | Low | 1.4% (0.7%, 2.0%) | 1.3% (0.6%, 2.0%) | 2.2% (0.7%, 3.7%) | 3.1% (1.1%, 5.1%) | 3.1% (0.4%, 5.8%) |
|  | Middle | 23.0% (20.5%, 25.5%) | 22.7% (20.2%, 25.1%) | 30.5% (25.8%, 35. 3%) | 44.7% (38.9%, 50.4%) | 50.9% (43.1%, 58.7%) |
|  | High | 75.6% (73.1%, 78.2%) | 76.1% (73.5%, 78.6%) | 67.3% (62.5%, 72.1%) | 52.2% (46.5%, 58.0%) | 46.0% (38.2%, 53.7%) |
| Portugal | n | 621 | 607 | 192 | 41 | 25 |
|  | Sex |  |  |  |  |  |
|  | Female | 66.0% (62.3%, 69.8%) | 65.6% (61.8%, 69.4%) | 68.2% (61.6%, 74.9%) | 73.2% (59.0%, 87.3%) | 72.0% (53.1%, 90.9%) |
|  | Male | 34.0% (30.2%, 37.7%) | 34.4% (30.6%, 38.2%) | 31.8% (25.1%, 38.4%) | 26.8% (12.7%, 41.0%) | 28.0% (9.1%, 46.9%) |
|  | Other | - | - | - | - | - |
|  | Age group |  |  |  |  |  |
|  | 18-34 | 15.0% (12.2%, 17.8%) | 15.2% (12.3%, 18.0%) | 20.3% (14.6%, 26.1%) | 24.4% (10.7%, 38.1%) | 20.0% (3.1%, 36.9%) |
|  | 35-54 | 58.9% (55.1%, 62.8%) | 58.8% (54.9%, 62.7%) | 62.5% (55.6%, 69.4%) | 68.3% (53.4%, 83.2%) | 68.0% (48.3%, 87.7%) |
|  | 55+ | 26.1% (22.6%, 29.6%) | 26.0% (22.5%, 29.5%) | 17.2% (11.8%, 22.6%) | 7.3% (0.0%, 15.6%) | 12.0% (0.0%, 25.7%) |
|  | Education |  |  |  |  |  |
|  | Low | 0.3% (0.0%, 0.8%) | 0.3% (0.0%, 0.8%) | - | - | - |
|  | Middle | 8.9% (6.6%, 11.1%) | 8.9% (6.6%, 11.2%) | 10.9% (6.5%, 15.4%) | 4.9% (0.0%, 11.8%) | 8.0% (0.0%, 19.4%) |
|  | High | 90.8% (88.5%, 93.1%) | 90.8% (88.5%, 93.1%) | 89.1% (84.6%, 93.5%) | 95.1% (88.2%, 100.0%) | 92.0% (80.6%, 100.0%) |
| Russia | n | 724 | 702 | 271 | 58 | 35 |
|  | Sex |  |  |  |  |  |
|  | Female | 60.8% (57.2%, 64.3%) | 61.1% (57.5%, 64.7%) | 53.9% (47.9%, 59.8%) | 39.7% (26.7%, 52.6%) | 34.3% (17.7%, 50.8%) |
|  | Male | 38.7% (35.1%, 42.2%) | 38.3% (34.7%, 41.9%) | 44.6% (38.7%, 50.6%) | 56.9% (43.8%, 70.0%) | 62.9% (46.0%, 79.7%) |
|  | Other | 0.6% (0.0%, 1.1%) | 0.6% (0.0%, 1.1%) | 1.5% (0.0%, 2.9%) | 3.4% (0.0%, 8.3%) | 2.9% (0.0%, 8.7%) |
|  | Age group |  |  |  |  |  |
|  | 18-34 | 44.8% (41.1%, 48.4%) | 44.6% (40.9%, 48.3%) | 51.7% (45.7%, 57.6%) | 60.3% (47.4%, 73.3%) | 62.9% (46.0%, 79.7%) |
|  | 35-54 | 40.9% (37.3%, 44.5%) | 41.2% (37.5%, 44.8%) | 38.4% (32.5%, 44.2%) | 31.0% (18.8%, 43.3%) | 28.6% (12.8%, 44.3%) |
|  | 55+ | 14.4% (11.8%, 16.9%) | 14.2% (11.7%, 16.8%) | 10.0% (6.4%, 13.6%) | 8.6% (1.2%, 16.1%) | 8.6% (0.0%, 18.3%) |
|  | Education |  |  |  |  |  |
|  | Low | 1.8% (0.8%, 2.8%) | 1.6% (0.6%, 2.5%) | 4.1% (1.7%, 6.4%) | 6.9% (0.2%, 13.6%) | 8.6% (0.0%, 18.3%) |
|  | Middle | 12.7% (10.3%, 15.1%) | 12.5% (10.1%, 15.0%) | 17.7% (13.1%, 22.3%) | 20.7% (9.9%, 31.4%) | 20.0% (6.1%, 33.9%) |
|  | High | 85.5% (82.9%, 88.1%) | 85.9% (83.3%, 88.5%) | 78.2% (73.3%, 83.2%) | 72.4% (60.6%, 84.3%) | 71.4% (55.7%, 87.2%) |
| Slovakia | n | 466 | 453 | 159 | 71 | 39 |
|  | Sex |  |  |  |  |  |
|  | Female | 66.7% (62.4%, 71.0%) | 66.9% (62.5%, 71.2%) | 57.9% (50.1%, 65.6%) | 46.5% (34.6%, 58.4%) | 43.6% (27.3%, 59.9%) |
|  | Male | 33.3% (29.0%, 37.6%) | 33.1% (28.8%, 37.5%) | 42.1% (34.4%, 49.8%) | 53.5% (41.6%, 65.4%) | 56.4% (40.1%, 72.7%) |
|  | Other | - | - | - | - | - |
|  | Age group |  |  |  |  |  |
|  | 18-34 | 58.8% (54.3%, 63.3%) | 59.4% (54.8%, 63.9%) | 64.8% (57.3%, 72.3%) | 70.4% (59.5%, 81.3%) | 56.4% (40.1%, 72.7%) |
|  | 35-54 | 33.3% (29.0%, 37.6%) | 33.1% (28.8%, 37.5%) | 28.9% (21.8%, 36.1%) | 21.1% (11.4%, 30.9%) | 33.3% (17.9%, 48.8%) |
|  | 55+ | 7.9% (5.5%, 10.4%) | 7.5% (5.1%, 9.9%) | 6.3% (2.5%, 10.1%) | 8.5% (1.8%, 15.1%) | 10.3% (0.3%, 20.2%) |
|  | Education |  |  |  |  |  |
|  | Low | 2.1% (0.8%, 3.5%) | 2.0% (0.7%, 3.3%) | 4.4% (1.2%, 7.6%) | 5.6% (0.1%, 11.1%) | 2.6% (0.0%, 7.8%) |
|  | Middle | 28.8% (24.6%, 32.9%) | 29.1% (24.9%, 33.3%) | 35.8% (28.3%, 43.4%) | 31.0% (20.0%, 42.0%) | 33.3% (17.9%, 48.8%) |
|  | High | 69.1% (64.9%, 73.3%) | 68.9% (64.6%, 73.2%) | 59.7% (52.0%, 67.5%) | 63.4% (51.9%, 74.9%) | 64.1% (48.3%, 79.9%) |
| Slovenia | n | 512 | 500 | 136 | 45 | 24 |
|  | Sex |  |  |  |  |  |
|  | Female | 73.8% (70.0%, 77.6%) | 74.0% (70.1%, 77.8%) | 68.4% (60.5%, 76.3%) | 53.3% (38.2%, 68.5%) | 62.5% (41.6%, 83.4%) |
|  | Male | 25.6% (21.8%, 29.4%) | 25.4% (21.6%, 29.2%) | 30.9% (23.0%, 38.7%) | 44.4% (29.3%, 59.5%) | 33.3% (13.0%, 53.7%) |
|  | Other | 0.6% (0.0%, 1.2%) | 0.6% (0.0%, 1.3%) | 0.7% (0.0%, 2.2%) | 2.2% (0.0%, 6.7%) | 4.2% (0.0%, 12.8%) |
|  | Age group |  |  |  |  |  |
|  | 18-34 | 27.9% (24.0%, 31.8%) | 28.2% (24.2%, 32.2%) | 29.4% (21.7%, 37.2%) | 44.4% (29.3%, 59.5%) | 58.3% (37.1%, 79.6%) |
|  | 35-54 | 52.0% (47.6%, 56.3%) | 52.4% (48.0%, 56.8%) | 50.0% (41.5%, 58.5%) | 37.8% (23.0%, 52.5%) | 20.8% (3.3%, 38.4%) |
|  | 55+ | 20.1% (16.6%, 23.6%) | 19.4% (15.9%, 22.9%) | 20.6% (13.7%, 27.5%) | 17.8% (6.2%, 29.4%) | 20.8% (3.3%, 38.4%) |
|  | Education |  |  |  |  |  |
|  | Low | 0.8% (0.0%, 1.5%) | 0.8% (0.0%, 1.6%) | 1.5% (0.0%, 3.5%) | 4.4% (0.0%, 10.7%) | 8.3% (0.0%, 20.3%) |
|  | Middle | 16.4% (13.2%, 19.6%) | 16.4% (13.1%, 19.7%) | 19.9% (13.1%, 26.6%) | 26.7% (13.2%, 40.1%) | 20.8% (3.3%, 38.4%) |
|  | High | 82.8% (79.5%, 86.1%) | 82.8% (79.5%, 86.1%) | 78.7% (71.7%, 85.6%) | 68.9% (54.8%, 83.0%) | 70.8% (51.2%, 90.4%) |
| Spain | n | 2,860 | 2,773 | 927 | 271 | 167 |
|  | Sex |  |  |  |  |  |
|  | Female | 64.0% (62.3%, 65.8%) | 63.8% (62.0%, 65.6%) | 66.1% (63.1%, 69.2%) | 56.1% (50.1%, 62.0%) | 50.3% (42.6%, 58.0%) |
|  | Male | 35.8% (34.1%, 37.6%) | 36.0% (34.2%, 37.8%) | 33.7% (30.6%, 36.7%) | 43.2% (37.2%, 49.1%) | 48.5% (40.8%, 56.2%) |
|  | Other | 0.1% (0.0%, 0.3%) | 0.1% (0.0%, 0.3%) | 0.2% (0.0%, 0.5%) | 0.7% (0.0%, 1.8%) | 1.2% (0.0%, 2.9%) |
|  | Age group |  |  |  |  |  |
|  | 18-34 | 32.0% (30.2%, 33.7%) | 32.3% (30.6%, 34.1%) | 34.6% (31.6%, 37.7%) | 46.9% (40.9%, 52.8%) | 37.7% (30.3%, 45.2%) |
|  | 35-54 | 52.4% (50.5%, 54.2%) | 52.1% (50.3%, 54.0%) | 51.6% (48.3%, 54.8%) | 43.2% (37.2%, 49.1%) | 53.3% (45.6%, 60.9%) |
|  | 55+ | 15.7% (14.3%, 17.0%) | 15.5% (14.2%, 16.9%) | 13.8% (11.6%, 16.0%) | 10.0% (6.4%, 13.6%) | 9.0% (4.6%, 13.4%) |
|  | Education |  |  |  |  |  |
|  | Low | 2.4% (1.8%, 2.9%) | 2.2% (1.7%, 2.7%) | 3.5% (2.3%, 4.6%) | 4.1% (1.7%, 6.4%) | 3.0% (0.4%, 5.6%) |
|  | Middle | 13.5% (12.2%, 14.7%) | 13.0% (11.7%, 14.2%) | 18.9% (16.4%, 21.4%) | 13.3% (9.2%, 17.4%) | 16.2% (10.5%, 21.8%) |
|  | High | 84.1% (82.8%, 85.5%) | 84.8% (83.5%, 86.2%) | 77.7% (75.0%, 80.4%) | 82.7% (78.1%, 87.2%) | 80.8% (74.8%, 86.9%) |
| Sweden | n | 733 | 721 | 115 | 32 | 23 |
|  | Sex |  |  |  |  |  |
|  | Female | 79.9% (77.0%, 82.9%) | 80.0% (77.1%, 83.0%) | 75.7% (67.7%, 83.6%) | 62.5% (44.8%, 80.2%) | 56.5% (34.6%, 78.4%) |
|  | Male | 19.9% (17.0%, 22.8%) | 19.8% (16.9%, 22.8%) | 24.3% (16.4%, 32.3%) | 37.5% (19.8%, 55.2%) | 43.5% (21.6%, 65.4%) |
|  | Other | 0.1% (0.0%, 0.4%) | 0.1% (0.0%, 0.4%) | - | - | - |
|  | Age group |  |  |  |  |  |
|  | 18-34 | 18.7% (15.9%, 21.5%) | 18.6% (15.7%, 21.4%) | 21.7% (14.1%, 29.4%) | 34.4% (17.0%, 51.8%) | 47.8% (25.7%, 69.9%) |
|  | 35-54 | 46.5% (42.9%, 50.1%) | 46.7% (43.1%, 50.4%) | 53.9% (44.7%, 63.2%) | 56.2% (38.1%, 74.4%) | 39.1% (17.6%, 60.7%) |
|  | 55+ | 34.8% (31.3%, 38.2%) | 34.7% (31.2%, 38.2%) | 24.3% (16.4%, 32.3%) | 9.4% (0.0%, 20.1%) | 13.0% (0.0%, 27.9%) |
|  | Education |  |  |  |  |  |
|  | Low | 1.0% (0.2%, 1.7%) | 1.0% (0.3%, 1.7%) | 0.9% (0.0%, 2.6%) | 3.1% (0.0%, 9.5%) | - |
|  | Middle | 16.0% (13.3%, 18.6%) | 15.1% (12.5%, 17.7%) | 27.8% (19.5%, 36.1%) | 18.8% (4.5%, 33.0%) | 17.4% (0.6%, 34.2%) |
|  | High | 83.1% (80.4%, 85.8%) | 83.9% (81.2%, 86.6%) | 71.3% (62.9%, 79.7%) | 78.1% (63.0%, 93.3%) | 82.6% (65.8%, 99.4%) |
| Ukraine | n | 487 | 478 | 173 | 53 | 23 |
|  | Sex |  |  |  |  |  |
|  | Female | 64.3% (60.0%, 68.5%) | 64.2% (59.9%, 68.5%) | 55.5% (48.0%, 63.0%) | 50.9% (37.0%, 64.9%) | 60.9% (39.3%, 82.4%) |
|  | Male | 35.5% (31.3%, 39.8%) | 35.6% (31.3%, 39.9%) | 44.5% (37.0%, 52.0%) | 49.1% (35.1%, 63.0%) | 39.1% (17.6%, 60.7%) |
|  | Other | 0.2% (0.0%, 0.6%) | 0.2% (0.0%, 0.6%) | - | - | - |
|  | Age group |  |  |  |  |  |
|  | 18-34 | 50.5% (46.1%, 55.0%) | 50.2% (45.7%, 54.7%) | 56.6% (49.2%, 64.1%) | 77.4% (65.7%, 89.0%) | 78.3% (60.0%, 96.5%) |
|  | 35-54 | 38.4% (34.1%, 42.7%) | 38.5% (34.1%, 42.9%) | 38.2% (30.8%, 45.5%) | 17.0% (6.5%, 27.4%) | 13.0% (0.0%, 27.9%) |
|  | 55+ | 11.1% (8.3%, 13.9%) | 11.3% (8.4%, 14.1%) | 5.2% (1.9%, 8.5%) | 5.7% (0.0%, 12.1%) | 8.7% (0.0%, 21.2%) |
|  | Education |  |  |  |  |  |
|  | Low | 1.4% (0.4%, 2.5%) | 1.5% (0.4%, 2.5%) | 2.3% (0.1%, 4.6%) | 1.9% (0.0%, 5.7%) | 4.3% (0.0%, 13.4%) |
|  | Middle | 17.2% (13.9%, 20.6%) | 16.9% (13.6%, 20.3%) | 15.0% (9.7%, 20.4%) | 34.0% (20.8%, 47.1%) | 34.8% (13.7%, 55.8%) |
|  | High | 81.3% (77.8%, 84.8%) | 81.6% (78.1%, 85.1%) | 82.7% (77.0%, 88.4%) | 64.2% (50.8%, 77.5%) | 60.9% (39.3%, 82.4%) |
| United Kingdom | n | 925 | 920 | 182 | 66 | 60 |
|  | Sex |  |  |  |  |  |
|  | Female | 64.8% (61.7%, 67.8%) | 64.8% (61.7%, 67.9%) | 61.0% (53.8%, 68.1%) | 57.6% (45.3%, 69.8%) | 46.7% (33.7%, 59.7%) |
|  | Male | 34.9% (31.8%, 38.0%) | 34.9% (31.8%, 38.0%) | 38.5% (31.3%, 45.6%) | 42.4% (30.2%, 54.7%) | 53.3% (40.3%, 66.3%) |
|  | Other | 0.3% (0.0%, 0.7%) | 0.3% (0.0%, 0.7%) | 0.5% (0.0%, 1.6%) | - | - |
|  | Age group |  |  |  |  |  |
|  | 18-34 | 24.8% (22.0%, 27.5%) | 24.8% (22.0%, 27.6%) | 31.9% (25.0%, 38.7%) | 54.5% (42.2%, 66.9%) | 51.7% (38.6%, 64.7%) |
|  | 35-54 | 43.4% (40.2%, 46.6%) | 43.4% (40.2%, 46.6%) | 44.5% (37.2%, 51.8%) | 31.8% (20.3%, 43.4%) | 36.7% (24.1%, 49.2%) |
|  | 55+ | 31.9% (28.9%, 34.9%) | 31.8% (28.8%, 34.9%) | 23.6% (17.4%, 29.9%) | 13.6% (5.1%, 22.1%) | 11.7% (3.3%, 20.0%) |
|  | Education |  |  |  |  |  |
|  | Low | 2.1% (1.1%, 3.0%) | 2.1% (1.1%, 3.0%) | 3.8% (1.0%, 6.7%) | - | - |
|  | Middle | 15.1% (12.8%, 17.4%) | 15.2% (12.9%, 17.5%) | 22.5% (16.4%, 28.7%) | 12.1% (4.0%, 20.2%) | 11.7% (3.3%, 20.0%) |
|  | High | 82.8% (80.4%, 85.2%) | 82.7% (80.3%, 85.2%) | 73.6% (67.2%, 80.1%) | 87.9% (79.8%, 96.0%) | 88.3% (80.0%, 96.7%) |
| Note. 95% CI = 95% confidence intervals. | | | | | | |

| **Appendix Table 2.** Reported changes for each substance for the entire survey sample | | | | | | |
| --- | --- | --- | --- | --- | --- | --- |
|  | Alcohol – Frequency | Alcohol – Quantity | Alcohol – HED | Tobacco | Cannabis | Other illicit substances |
|  | n=35,753 | | | n=9,816 | n=3,289 | n=1,961 |
|  | % (95% CI) | | | | | |
| Change in substance use | |  |  |  |  |  |
| Much more | 9.9% (6.4%, 13.5%) | 6.0% (2.4%, 9.6%) | 5.1% (1.4%, 8.7%) | 16.8% (11.5%, 22.1%) | 16.5% (7.6%, 25.5%) | 7.9% (-4.8%, 20.6%) |
| Slightly more | 20.2% (17.0%, 23.5%) | 14.2% (10.9%, 17.4%) | 10.9% (7.3%, 14.5%) | 24.3% (19.3%, 29.3%) | 18.4% (10.0%, 26.8%) | 14.2% (1.3%, 27.1%) |
| No change | 42.0% (39.1%, 44.8%) | 53.0% (50.4%, 55.6%) | 53.9% (51.4%, 56.4%) | 39.6% (34.7%, 44.5%) | 42.2% (34.7%, 49.8%) | 48.6% (39.0%, 58.2%) |
| Slightly less | 9.6% (6.4%, 12.9%) | 9.7% (6.3%, 13.1%) | 7.0% (3.8%, 10.3%) | 8.6% (2.4%, 14.7%) | 5.3% (-4.5%, 15.0%) | 7.7% (-8.6%, 24.0%) |
| Much less | 18.2% (14.8%, 21.6%) | 17.1% (13.7%, 20.6%) | 23.0% (19.7%, 26.4%) | 10.8% (4.8%, 16.7%) | 17.5% (8.0%, 27.1%) | 21.6% (11.1%, 32.2%) |
| Indicator of change | 2.3% (-1.9%, 6.7%) | -6.6% (-11.1%, -2.2%) | -14.0% (-18.7%, -9.4%) | 21.9% (14.7%, 29.0%) | 12.2% (0.4%, 24.1%) | -7.2% (-24.3%, 9.5%) |
| Note. HED = Heavy episodic drinking, 95% CI = 95% confidence intervals. | | | | | | |

| **Appendix Table 3.** Reported changes for each substance by country | | | | | | | |
| --- | --- | --- | --- | --- | --- | --- | --- |
| Country | Change in use | Alcohol – Frequency | Alcohol – Quantity | Alcohol –  HED | Tobacco | Cannabis | Other illicit substances |
|  |  | % (95% CI) or n | | | | | |
| Albania | n | 204 | | | 67 | 22 | 17 |
|  | Much more | 4.8% (-16.5%, 26.1%) | 4.8% (-16.7%, 26.2%) | 2.7% (-16.1%, 21.5%) | 18.4% (-11.3%, 48.2%) | 24.5% (-30.0%, 78.9%) | 8.2% (-29.8%, 46.3%) |
|  | Slightly more | 6.2% (-16.8%, 29.2%) | 6.8% (-15.2%, 28.8%) | 4.6% (-19.8%, 29.0%) | 27.0% (-4.1%, 58.1%) | 1.9% (-19.6%, 23.4%) | 2.0% (-25.3%, 29.2%) |
|  | No change | 48.2% (30.1%, 66.3%) | 52.9% (35.5%, 70.3%) | 51.4% (33.9%, 68.9%) | 30.2% (-1.6%, 62.0%) | 24.4% (-59.8%, 100.0%) | 44.5% (-31.2%, 100.0%) |
|  | Slightly less | 6.8% (-12.7%, 26.3%) | 5.3% (-13.5%, 24.0%) | 4.2% (-13.7%, 22.2%) | 6.4% (-27.3%, 40.1%) | 15.7% (-25.0%, 56.4%) | - |
|  | Much less | 34.0% (14.5%, 53.6%) | 30.2% (10.3%, 50.2%) | 37.1% (17.2%, 57.0%) | 18.0% (-15.7%, 51.7%) | 33.6% (-37.1%, 100.0%) | 45.3% (-29.2%, 100.0%) |
|  | Indicator of change | -29.9% (-57.0%, -1.8%) | -23.8% (-52.1%, 4.0%) | -34.0% (-63.2%, -5.4%) | 21.6% (-19.7%, 62.2%) | -22.7% (-97.1%, 51.8%) | -35.8% (-100.0%, 49.1%) |
| Czechia | n | 1,434 | | | 456 | 197 | 101 |
|  | Much more | 7.3% (-0.7%, 15.3%) | 3.8% (-4.2%, 11.9%) | 4.4% (-4.8%, 13.6%) | 12.9% (0.1%, 25.7%) | 9.3% (-12.4%, 31.0%) | 5.8% (-14.9%, 26.6%) |
|  | Slightly more | 19.6% (11.6%, 27.5%) | 14.9% (5.9%, 23.9%) | 12.4% (2.7%, 22.0%) | 24.4% (11.8%, 37.0%) | 15.4% (-5.0%, 35.7%) | 3.9% (-19.6%, 27.4%) |
|  | No change | 43.5% (36.0%, 50.9%) | 56.6% (50.2%, 63.0%) | 53.8% (47.4%, 60.3%) | 47.4% (35.4%, 59.5%) | 57.2% (38.8%, 75.6%) | 72.9% (51.7%, 94.2%) |
|  | Slightly less | 15.1% (6.0%, 24.2%) | 12.4% (3.8%, 21.0%) | 10.0% (1.3%, 18.6%) | 8.5% (-7.6%, 24.5%) | 4.6% (-9.8%, 19.0%) | 2.7% (-23.8%, 29.2%) |
|  | Much less | 14.6% (6.1%, 23.0%) | 12.2% (4.0%, 20.4%) | 19.4% (11.1%, 27.8%) | 6.8% (-5.4%, 19.1%) | 13.5% (-5.1%, 32.1%) | 14.6% (-16.4%, 45.6%) |
|  | Indicator of change | -2.9% (-13.4%, 8.0%) | -5.8% (-17.4%, 5.5%) | -12.6% (-24.7%, -0.8%) | 22.2% (4.2%, 39.9%) | 6.5% (-19.1%, 32.0%) | -7.9% (-45.4%, 29.2%) |
| Denmark | n | 2,437 | | | 710 | 247 | 113 |
|  | Much more | 5.7% (-1.6%, 13.0%) | 2.9% (-4.4%, 10.2%) | 4.0% (-3.3%, 11.2%) | 16.1% (2.5%, 29.7%) | 14.3% (-5.5%, 34.0%) | 6.2% (-19.1%, 31.4%) |
|  | Slightly more | 25.2% (18.7%, 31.6%) | 15.0% (8.0%, 22.0%) | 11.6% (4.7%, 18.5%) | 26.5% (15.0%, 38.1%) | 30.3% (14.5%, 46.0%) | 23.6% (-1.4%, 48.6%) |
|  | No change | 39.3% (33.0%, 45.6%) | 55.3% (50.2%, 60.4%) | 49.7% (44.1%, 55.3%) | 33.1% (21.8%, 44.5%) | 38.8% (20.9%, 56.7%) | 38.4% (13.9%, 62.8%) |
|  | Slightly less | 16.1% (10.3%, 21.8%) | 16.6% (10.2%, 23.0%) | 13.9% (7.7%, 20.1%) | 13.7% (0.3%, 27.2%) | 9.0% (-9.9%, 27.9%) | 12.9% (-15.1%, 40.8%) |
|  | Much less | 13.8% (7.8%, 19.8%) | 10.2% (3.7%, 16.8%) | 20.9% (14.8%, 27.0%) | 10.5% (-1.7%, 22.8%) | 7.7% (-9.8%, 25.1%) | 19.0% (-8.8%, 46.9%) |
|  | Indicator of change | 1.0% (-6.9%, 9.1%) | -8.9% (-18.0%, 0.0%) | -19.2% (-28.0%, -10.7%) | 18.5% (2.4%, 34.5%) | 27.9% (5.5%, 50.7%) | -2.4% (-37.6%, 32.6%) |
| Finland | n | 3,471 | | | 966 | 227 | 123 |
|  | Much more | 5.9% (0.4%, 11.4%) | 2.7% (-2.0%, 7.4%) | 2.5% (-2.9%, 7.9%) | 11.4% (2.5%, 20.2%) | 11.8% (-7.8%, 31.5%) | 20.6% (-7.6%, 48.8%) |
|  | Slightly more | 12.6% (8.1%, 17.2%) | 9.8% (4.5%, 15.1%) | 7.3% (2.1%, 12.5%) | 23.8% (15.2%, 32.5%) | 17.2% (-1.0%, 35.4%) | 14.5% (-14.7%, 43.8%) |
|  | No change | 44.4% (40.5%, 48.2%) | 56.7% (53.3%, 60.1%) | 56.0% (52.6%, 59.3%) | 38.8% (31.0%, 46.5%) | 51.5% (38.1%, 64.9%) | 44.3% (26.0%, 62.6%) |
|  | Slightly less | 15.9% (11.1%, 20.6%) | 14.1% (9.3%, 18.9%) | 10.1% (4.7%, 15.6%) | 9.9% (0.7%, 19.1%) | 7.7% (-10.5%, 26.0%) | 3.0% (-13.3%, 19.3%) |
|  | Much less | 21.2% (16.6%, 25.8%) | 16.7% (12.0%, 21.5%) | 24.1%, 19.6%, 28.6%) | 16.2% (6.9%, 25.4%) | 11.7% (-4.1%, 27.6%) | 17.6% (-6.8%, 42.0%) |
|  | Indicator of change | -18.6% (-24.5%, -12.4%) | -18.3% (-25.0%, -11.8%) | -24.4% (-31.1%, -17.8%) | 9.3% (-2.4%, 20.8%) | 9.6% (-13.8%, 32.8%) | 14.2% (-20.7%, 49.1%) |
| France | n | 314 | | | 137 | 65 | 40 |
|  | Much more | 11.7% (-5.0%, 28.4%) | 5.8% (-11.8%, 23.5%) | 5.4% (-11.0%, 21.8%) | 20.2% (1.3%, 41.7%) | 19.3% (-5.4%, 44.0%) | 14.8% (-22.4%, 52.0%) |
|  | Slightly more | 21.3% (8.9%, 33.7%) | 16.7% (3.5%, 29.8%) | 12.5% (-2.5%, 27.5%) | 30.3% (11.9%, 48.7%) | 13.2% (-17.6%, 44.0%) | 11.9% (-33.0%, 56.8%) |
|  | No change | 41.9% (28.6%, 55.2%) | 51.3% (39.7%, 63.0%) | 49.9% (38.6%, 61.2%) | 34.0% (14.7%, 53.2%) | 32.7% (10.1%, 55.2%) | 36.5% (6.6%, 66.3%) |
|  | Slightly less | 6.6% (-5.4%, 18.7%) | 9.2% (-5.4%, 23.9%) | 8.0% (-6.8%, 22.8%) | 4.9% (-19.0%, 28.9%) | 4.9% (-20.5%, 30.3%) | 5.8% (-40.0%, 51.6%) |
|  | Much less | 18.5% (4.4%, 32.6%) | 16.9% (2.5%, 31.2%) | 24.3% (9.8%, 38.8%) | 10.6% (-11.5%, 32.7%) | 30.0% (-0.9%, 60.9%) | 31.1% (0.9%, 61.2%) |
|  | Indicator of change | 7.8% (-9.5%, 25.8%) | -3.5% (-22.6%, 15.3%) | -14.2% (-34.4%, 5.4%) | 35.3% (8.2%, 62.3%) | -2.4% (-39.7%, 35.5%) | -10.5% (-59.6%, 38.4%) |
| Germany | n | 1,517 | | | 588 | 333 | 188 |
|  | Much more | 12.1% (6.0%, 18.1%) | 8.0% (1.8%, 14.3%) | 5.7% (-0.7%, 12.1%) | 20.3% (11.6%, 29.1%) | 17.0% (4.6%, 29.5%) | 6.1% (-12.0%, 24.2%) |
|  | Slightly more | 20.6% (15.0%, 26.2%) | 17.4% (11.6%, 23.3%) | 10.7% (4.7%, 16.7%) | 27.9% (19.2%, 36.5%) | 28.5% (16.8%, 40.1%) | 13.9% (-4.9%, 32.6%) |
|  | No change | 42.9% (37.7%, 48.1%) | 53.1% (48.5%, 57.7%) | 53.5% (48.9%, 58.1%) | 39.0% (30.6%, 47.4%) | 40.5% (29.7%, 51.4%) | 50.1% (36.8%, 63.3%) |
|  | Slightly less | 11.9% (5.8%, 17.9%) | 10.6% (4.8%, 16.4%) | 9.6% (3.6%, 15.6%) | 7.2% (-2.2%, 16.5%) | 5.8% (-6.2%, 17.7%) | 8.1% (-8.4%, 24.6%) |
|  | Much less | 12.5% (6.5%, 18.5%) | 10.8% (4.7%, 16.9%) | 20.4% (14.7%, 26.0%) | 5.7% (-2.8%, 14.2%) | 8.2% (-3.8%, 20.3%) | 21.9% (6.9%, 36.8%) |
|  | Indicator of change | 8.3% (1.0%, 15.9%) | 4.1% (-3.8%, 11.8%) | -13.5% (-21.5%, -5.7%) | 35.5% (24.1%, 46.7%) | 31.5% (16.3%, 46.9%) | -10.2% (-33.1%, 12.8%) |
| Greece | n | 531 | | | 248 | 84 | 38 |
|  | Much more | 10.8% (-7.8%, 29.3%) | 9.2% (-10.4%, 28.9%) | 8.1% (-11.2%, 27.5%) | 36.1% (15.6%, 56.5%) | 34.2% (-10.4%, 78.8%) | - |
|  | Slightly more | 13.9% (-0.4%, 28.2%) | 12.3% (-2.6%, 27.1%) | 9.6% (-8.1%, 27.3%) | 21.4% (2.2%, 40.7%) | 10.0% (-17.3%, 37.4%) | 3.7% (-15.0%, 22.5%) |
|  | No change | 37.2% (25.0%, 49.4%) | 46.6% (35.8%, 57.3%) | 51.2% (40.8%, 61.6%) | 26.0% (4.4%, 47.5%) | 28.0% (-10.1%, 66.1%) | 96.3% (79.7%, 100.0%) |
|  | Slightly less | 9.9% (-4.1%, 23.9%) | 11.2% (-2.9%, 25.3%) | 8.1% (-5.1%, 21.3%) | 6.3% (-11.1%, 23.7%) | 6.1% (-19.9%, 32.0%) | - |
|  | Much less | 28.2% (16.9%, 39.5%) | 20.8% (8.5%, 33.0%) | 22.9% (11.0%, 34.8%) | 10.3% (-6.8%, 27.3%) | 21.7% (-7.6%, 51.1%) | - |
|  | Indicator of change | -13.5% (-31.5%, 5.0%) | -10.3% (-30.1%, 9.3%) | -13.2% (-34.1%, 7.5%) | 41.3% (18.3%, 64.0%) | 16.4% (-29.1%, 62.4%) | 3.5% (-15.2%, 22.7%) |
| Hungary | n | 434 | | | 189 | 68 | 39 |
|  | Much more | 3.1% (-9.9%, 16.1%) | 0.8% (-9.3%, 10.8%) | 0.3% (-7.9%, 8.4%) | 11.1% (-10.5%, 32.6%) | 6.3% (-31.2%, 43.8%) | 7.7% (-44.6%, 60.1%) |
|  | Slightly more | 9.2% (-1.4%, 19.8%) | 8.2% (-4.2%, 20.7%) | 5.6% (-7.5%, 18.7%) | 47.8% (33.8%, 61.9%) | 18.9% (-16.5%, 54.4%) | 15.4% (-24.9%, 55.7%) |
|  | No change | 69.1% (61.5%, 76.6%) | 75.2% (68.6%, 81.8%) | 76.1% (69.5%, 82.7%) | 29.2% (14.1%, 44.2%) | 37.0% (13.1%, 60.9%) | 41.4% (6.6%, 76.1%) |
|  | Slightly less | 6.8% (-5.0%, 18.6%) | 8.6% (-3.6%, 20.7%) | 4.5% (-6.9%, 15.9%) | 7.8% (-15.0%, 30.5%) | 7.0% (-15.6%, 29.6%) | 8.6% (-42.6%, 59.9%) |
|  | Much less | 11.8% (-0.2%, 23.9%) | 7.3% (-5.7%, 20.2%) | 13.5% (1.6%, 25.5%) | 4.2% (-12.9%, 21.2%) | 30.8% (1.7%, 59.8%) | 26.9% (-14.0%, 67.7%) |
|  | Indicator of change | -6.4% (-21.6%, 9.3%) | -6.7% (-23.9%, 10.1%) | -12.2% (-29.5%, 4.8%) | 47.3% (23.2%, 71.2%) | -12.5% (-54.6%, 29.9%) | -12.7% (-71.8%, 45.7%) |
| Iceland | n | 477 | | | 107 | 14 | 12 |
|  | Much more | 8.8% (-12.3%, 29.9%) | 3.4% (-20.2%, 26.9%) | 0.9% (-10.4%, 12.1%) | 5.4% (-9.8%, 20.6%) | 50.8% (-13.1%, 100.0%) | 57.6% (-39.2%, 100.0%) |
|  | Slightly more | 11.0% (-3.9%, 26.0%) | 11.1% (-7.9%, 30.1%) | 6.6% (-12.3%, 25.6%) | 29.6% (-0.1%, 59.2%) | 9.4% (-47.8%, 66.7%) | 14.2% (-40.4%, 68.8%) |
|  | No change | 45.5% (33.8%, 57.3%) | 61.5% (50.9%, 72.1%) | 56.5% (44.9%, 68.1%) | 52.4% (25.5%, 79.4%) | 28.2% (-45.4%, 100.0%) | 15.9% (-28.6%, 60.4%) |
|  | Slightly less | 14.6% (-4.3%, 33.5%) | 10.6% (-4.7%, 26.0%) | 6.5% (-12.3%, 25.2%) | 6.6% (-12.7%, 25.9%) | - | - |
|  | Much less | 20.0% (4.5%, 35.5%) | 13.4% (-1.4%, 28.2%) | 29.6% (15.4%, 43.8%) | 6.0% (-10.5%, 22.5%) | 11.6% (-24.0%, 47.2%) | 12.3% (-52.1%, 76.7%) |
|  | Indicator of change | -14.9% (-36.6%, 7.8%) | -9.5% (-33.5%, 14.2%) | -28.5% (-51.5%, -5.8%) | 22.9% (-9.6%, 54.6%) | 48.6% (-15.9%, 100.0%) | 58.7% (-39.9%, 100.0%) |
| Ireland | n | 483 | | | 92 | 30 | 17 |
|  | Much more | 11.1% (-5.5%, 27.6%) | 6.0% (-14.2%, 26.2%) | 7.3% (-11.2%, 25.8%) | 28.0% (-7.5%, 63.5%) | 5.9% (-27.1%, 39.0%) | 37.7% (-46.4%, 100.0%) |
|  | Slightly more | 28.2% (13.6%, 42.8%) | 22.8% (7.0%, 38.6%) | 14.6% (-1.9%, 31.0%) | 22.8% (-7.1%, 52.7%) | 47.2% (-6.8%, 100.0%) | - |
|  | No change | 31.5% (13.9%, 49.1%) | 38.2% (22.2%, 54.1%) | 45.1% (30.5%, 59.8%) | 25.8% (-6.7%, 58.4%) | 39.8% (0.8%, 78.7%) | 15.6% (-21.1%, 52.4%) |
|  | Slightly less | 12.5% (-4.8%, 29.8%) | 13.6% (-0.3%, 27.5%) | 7.3% (-7.1%, 21.6%) | 6.2% (-30.8%, 43.3%) | 3.1% (-17.3%, 23.5%) | - |
|  | Much less | 16.8% (-0.1%, 33.7%) | 19.5% (2.1%, 36.9%) | 25.7% (9.1%, 42.3%) | 17.2% (-25.5%, 59.9%) | 4.0% (-34.3%, 42.2%) | 46.6% (-7.7%, 100.0%) |
|  | Indicator of change | 9.8% (-10.0%, 30.5%) | -4.2% (-25.6%, 16.7%) | -10.9% (-33.4%, 10.8%) | 28.0% (-19.6%, 75.4%) | 46.0% (-10.7, 100.0%) | -9.8% (-100.0%, 92.4%) |
| Italy | n | 687 | | | 289 | 98 | 52 |
|  | Much more | 7.8% (-5.7%, 21.2%) | 6.4% (-8.0%, 20.8%) | 3.5% (-12.1%, 19.0%) | 18.6% (1.1%, 36.2%) | 8.7% (-28.8%, 46.1%) | - |
|  | Slightly more | 16.0% (4.8%, 27.2%) | 12.8% (2.4%, 23.2%) | 4.5% (-10.3%, 19.3%) | 31.1% (15.5%, 46.6%) | 24.1% (-14.6%, 62.8%) | 1.3% (-20.8%, 23.4%) |
|  | No change | 37.0% (27.1%, 47.0%) | 40.8% (31.6%, 50.0%) | 56.2% (48.2%, 64.2%) | 29.4% (13.8%, 45.1%) | 31.5% (12.2%, 50.7%) | 74.0% (46.5%, 100.0%) |
|  | Slightly less | 7.8% (-6.4%, 22.1%) | 9.7% (-5.1%, 24.5%) | 1.8% (-7.0%, 10.5%) | 7.7% (-10.4%, 25.8%) | 1.2% (-10.0%, 12.4%) | - |
|  | Much less | 31.4% (19.1%, 43.8%) | 30.4% (17.5%, 43.2%) | 34.0% (20.9%, 47.2%) | 13.2% (-5.3%, 31.7%) | 34.6% (3.4%, 65.8%) | 24.8% (-27.9%, 77.4%) |
|  | Indicator of change | -15.6% (-31.1%, 0.5%) | -20.8% (-37.1%, -4.6%) | -27.8% (-47.5%, -8.5%) | 29.1% (7.1%, 50.9%) | -2.9% (-49.1%, 43.0%) | -24.1% (-81.7%, 34.7%) |
| Norway | n | 15,520 | | | 3,445 | 976 | 664 |
|  | Much more | 5.6% (3.5%, 7.6%) | 2.6% (0.1%, 5.0%) | 2.3% (-0.2%, 4.8%) | 12.7% (8.3%, 17.0%) | 12.8% (4.5%, 21.2%) | 12.8% (2.3%, 23.2%) |
|  | Slightly more | 16.8% (14.8%, 18.8%) | 10.4% (8.4%, 12.4%) | 7.3% (5.1%, 9.5%) | 23.5% (19.3%, 27.8%) | 16.7% (9.3%, 24.1%) | 23.0% (13.6%, 32.3%) |
|  | No change | 48.8% (46.9%, 50.6%) | 60.1% (58.6%, 61.7%) | 57.9% (56.3%, 59.6%) | 46.5% (42.7%, 50.3%) | 40.7% (34.1%, 47.2%) | 40.9% (33.0%, 48.7%) |
|  | Slightly less | 14.2% (12.1%, 16.3%) | 15.1% (13.0%, 17.2%) | 11.6% (9.4%, 13.8%) | 7.8% (3.0%, 12.6%) | 8.9% (0.1%, 17.7%) | 6.7% (-2.8%, 16.2%) |
|  | Much less | 14.7% (12.5%, 16.9%) | 11.7% (9.5%, 14.0%) | 20.8% (18.7%, 22.9%) | 9.5% (5.1%, 13.9%) | 20.9% (13.4%, 28.4%) | 16.7% (7.8%, 25.6%) |
|  | Indicator of change | -6.6% (-9.2%, -3.8%) | -13.9% (-16.8%, -11.1%) | -22.8% (-25.8%, -19.9%) | 19.0% (13.1%, 24.7%) | -0.3% (-10.3%, 9.8%) | 12.3% (0.2%, 24.6%) |
| Poland | n | 1,090 | | | 367 | 291 | 161 |
|  | Much more | 8.4% (-4.0%, 20.8%) | 2.8% (-8.8%, 14.4%) | 3.1% (-10.3%, 16.5%) | 16.3% (-3.6%, 36.2%) | 23.6% (5.1%, 42.1%) | 13.1% (-12.9%, 39.2%) |
|  | Slightly more | 24.6% (13.1%, 36.1%) | 19.5% (6.7%, 32.3%) | 11.4% (-1.5%, 24.4%) | 22.8% (3.9%, 41.7%) | 12.4% (-5.6%, 30.5%) | 24.9% (0.4%, 49.4%) |
|  | No change | 35.4% (25.5%, 45.2%) | 47.2% (38.5%, 55.9%) | 50.1% (41.5%, 58.7%) | 35.0% (19.8%, 50.1%) | 39.0% (20.5%, 57.5%) | 36.4% (13.3%, 59.5%) |
|  | Slightly less | 13.6% (1.2%, 26.1%) | 12.3% (0.2%, 24.3%) | 8.4% (-4.0%, 20.8%) | 12.7% (-8.5%, 33.9%) | 6.1% (-17.1%, 29.2%) | 6.8% (-24.4%, 38.0%) |
|  | Much less | 18.0% (6.6%, 29.4%) | 18.3% (6.3%, 30.2%) | 27.0% (15.6%, 38.4%) | 13.2% (-5.0%, 31.4%) | 18.9% (-0.7%, 38.4%) | 18.7% (-5.7%, 43.2%) |
|  | Indicator of change | 1.3% (-13.2%, 16.5%) | -8.1% (-24.6%, 8.0%) | -20.7% (-37.4%, -4.4%) | 13.6% (-11.4%, 38.3%) | 11.1% (-13.8%, 36.5%) | 12.3% (-21.3%, 46.1%) |
| Portugal | n | 607 | | | 192 | 41 | 25 |
|  | Much more | 3.7% (-10.1%, 17.5%) | 2.2% (-13.6%, 18.0%) | 1.5% (-16.5%, 19.6%) | 18.2% (-11.7%, 48.2%) | 7.7% (-35.5%, 50.9%) | - |
|  | Slightly more | 12.9% (1.6%, 24.3%) | 11.5% (0.1%, 22.9%) | 4.6% (-6.5%, 15.7%) | 15.0% (-5.9%, 35.9%) | 11.1% (-25.6%, 47.9%) | - |
|  | No change | 60.6% (50.1%, 71.1%) | 65.7% (56.0%, 75.4%) | 71.5% (62.5%, 80.5%) | 41.5% (24.9%, 58.1%) | 68.2% (38.8%, 97.6%) | 37.4% (0.4%, 74.5%) |
|  | Slightly less | 10.1% (-6.7%, 26.9%) | 9.4% (-7.3%, 26.1%) | 3.8% (-5.2%, 12.9%) | 13.9% (-11.1%, 38.9%) | 12.3% (-33.2%, 57.9%) | 19.3% (-35.4%, 73.9%) |
|  | Much less | 12.6% (-3.3%, 28.4%) | 11.2% (-4.8%, 27.1%) | 18.6% (3.9%, 33.3%) | 11.3% (-16.1%, 38.8%) | 0.6% (-14.9%, 16.2%) | 43.3% (-18.7%, 100.0%) |
|  | Indicator of change | -6.1% (-24.6%, 13.0%) | -6.7% (-26.1%, 12.5%) | -16.2% (-35.3%, 2.2%) | 8.6% (-25.5%, 42.3%) | 5.9% (51.3%, 63.9%) | -62.0% (-100.0%, -15.4%) |
| Russia | n | 702 | | | 271 | 58 | 35 |
|  | Much more | 7.9% (-7.0%, 22.9%) | 5.4% (-11.7%, 22.6%) | 5.0% (-13.5%, 23.5%) | 12.8% (-4.8%, 30.5%) | 16.6% (-47.2%, 80.5%) | - |
|  | Slightly more | 20.1% (7.7%, 32.4%) | 14.3% (-1.8%, 30.4%) | 14.1% (-1.9%, 30.1%) | 16.2% (-3.8%, 36.2%) | 3.1% (-16.7%, 22.9%) | 9.5% (-48.1%, 67.2%) |
|  | No change | 48.5% (37.0%, 59.9%) | 58.1% (48.7%, 67.4%) | 55.0% (45.3%, 64.6%) | 51.4% (33.3%, 69.6%) | 36.8% (12.0%, 61.6%) | 40.9% (9.6%, 72.1%) |
|  | Slightly less | 8.2% (-6.6%, 23.0%) | 5.7% (-3.7%, 15.0%) | 3.4% (-7.3%, 14.0%) | 8.9% (-11.7%, 29.4%) | 16.9% (-46.5%, 80.3%) | 42.5% (-35.6%, 100.0%) |
|  | Much less | 15.3% (3.3%, 27.4%) | 16.5% (2.1%, 30.9%) | 22.5% (9.0%, 36.0%) | 10.7% (-11.6%, 33.0%) | 26.6% (-33.6%, 86.8%) | 7.0% (-22.2%, 36.2%) |
|  | Indicator of change | 4.4% (-12.1%, 21.6%) | -2.4% (-22.6%, 17.5%) | -6.7% (-27.2%, 13.5%) | 9.9% (-17.1%, 36.4%) | -23.7% (-100.0%, 53.8%) | -40.6% (-100.0%, 50.2%) |
| Slovakia | n | 453 | | | 159 | 71 | 39 |
|  | Much more | 7.8% (-9.6%, 25.2%) | 4.1% (-13.4%, 21.6%) | 4.4% (-12.8%, 21.5%) | 5.8% (-12.8%, 24.3%) | 1.5% (-12.3%, 15.3%) | 2.1% (-17.6%, 21.8%) |
|  | Slightly more | 9.5% (-4.8%, 23.8%) | 10.2% (-6.0%, 26.4%) | 9.5% (-7.9%, 26.8%) | 20.9% (-3.4%, 45.3%) | 12.3% (-19.9%, 44.4%) | 17.4% (-34.0%, 68.9%) |
|  | No change | 45.4% (33.0%, 57.9%) | 53.5% (42.2%, 64.8%) | 54.0% (42.8%, 65.1%) | 49.7% (30.4%, 69.0%) | 52.9% (23.6%, 82.3%) | 64.2% (26.3%, 100.0%) |
|  | Slightly less | 11.1% (-4.3%, 26.6%) | 9.0% (-6.9%, 24.9%) | 8.7% (-7.0%, 24.3%) | 8.3% (-19.1%, 35.8%) | 7.3% (-33.9%, 48.5%) | 1.0% (-18.2%, 20.2%) |
|  | Much less | 26.1% (10.9%, 41.3%) | 23.2% (7.5%, 38.8%) | 23.5% (8.1%, 39.0%) | 15.3% (-9.5%, 40.1%) | 26.0% (-12.7%, 64.7%) | 15.4% (-39.5%, 70.3%) |
|  | Indicator of change | -20.0% (-39.5%, 0.3%) | -17.8% (-39.4%, 3.4%) | -18.4% (-40.5%, 3.3%) | 3.6% (-29.4%, 36.1%) | -19.5% (-66.1%, 27.9%) | 2.6% (-70.2%, 75.7%) |
| Slovenia | n | 500 | | | 136 | 45 | 24 |
|  | Much more | 3.9% (-16.2%, 24.0%) | 0.5% (-5.5%, 6.5%) | 0.2% (-5.8%, 6.3%) | 10.1% (-13.7%, 33.8%) | 21.4% (-18.8%, 61.6%) | - |
|  | Slightly more | 21.4% (6.0%, 36.7%) | 15.2% (-1.8%, 32.2%) | 9.2% (-8.5%, 27.0%) | 37.1% (10.6%, 63.6%) | 21.1% (-20.1%, 62.3%) | 3.3% (-22.8%, 29.4%) |
|  | No change | 49.1% (37.9%, 60.2%) | 61.2% (51.4%, 71.0%) | 60.6% (50.9%, 70.3%) | 36.9% (12.0%, 61.7%) | 32.4% (-2.1%, 67.0%) | 63.6% (24.1%, 100.0%) |
|  | Slightly less | 15.4% (-0.2%, 30.9%) | 14.9% (-1.2%, 30.9%) | 9.6% (-5.8%, 25.1%) | 5.2% (-19.5%, 29.8%) | - | - |
|  | Much less | 10.3% (-2.6%, 23.3%) | 8.3% (-3.7%, 20.3%) | 20.3% (4.4%, 36.2%) | 10.9% (-14.9%, 36.6%) | 25.0% (-29.5%, 79.6%) | 33.1% (-26.0%, 92.2%) |
|  | Indicator of change | -0.6% (-20.3%, 19.9%) | -7.3% (-29.2%, 14.0%) | -20.4% (-43.3%, 2.0%) | 31.7% (-2.3%, 65.1%) | 17.5% (-45.6%, 82.1%) | -30.5% (-96.1%, 36.0%) |
| Spain | n | 2,773 | | | 927 | 271 | 167 |
|  | Much more | 9.2% (2.0%, 16.3%) | 7.7% (0.6%, 14.7%) | 5.3% (-2.2%, 12.9%) | 18.7% (9.1%, 28.3%) | 12.1% (-7.7%, 31.9%) | 2.8% (-19.6%, 25.2%) |
|  | Slightly more | 19.4% (13.9%, 25.0%) | 17.1% (11.2%, 22.9%) | 10.2% (3.6%, 16.7%) | 30.4% (21.3%, 39.5%) | 25.6% (5.1%, 46.1%) | 18.1% (-7.4%, 43.7%) |
|  | No change | 32.7% (26.9%, 38.4%) | 36.7% (31.1%, 42.2%) | 43.3% (38.3%, 48.4%) | 31.9% (22.6%, 41.3%) | 45.6% (30.5%, 60.7%) | 46.6% (28.0%, 65.2%) |
|  | Slightly less | 11.0% (5.3%, 16.7%) | 8.8% (3.4%, 14.2%) | 7.2% (1.2%, 13.1%) | 10.1% (-0.4%, 20.7%) | 2.6% (-17.7%, 22.9%) | 3.8% (-21.6%, 29.1%) |
|  | Much less | 27.7% (22.1%, 33.3%) | 29.8% (24.3%, 35.3%) | 34.0% (28.7%, 39.3%) | 8.8% (-0.1%, 17.6%) | 14.1% (-2.0%, 30.1%) | 28.7% (2.4%, 55.0%) |
|  | Indicator of change | -10.1% (-17.4%, -2.6%) | -13.9% (-21.6%, -6.3%) | -25.7% (-34.0%, -17.5%) | 30.4% (18.3%, 42.3%) | 21.1% (-3.2%, 45.2%) | -11.8% (-47.7%, 23.7%) |
| Sweden | n | 721 | | | 115 | 32 | 23 |
|  | Much more | 1.0% (-8.1%, 10.2%) | 0.5% (-7.4%, 8.3%) | 0.2% (-9.5%, 10.0%) | 2.1% (-14.5%, 18.7%) | 7.3% (-32.2%, 46.8%) | - |
|  | Slightly more | 13.3% (-3.1%, 29.8%) | 9.0% (-9.1%, 27.1%) | 6.2% (-13.3%, 25.7%) | 21.9% (-14.7%, 58.4%) | 24.3% (-49.2%, 97.8%) | 4.1% (-34.8%, 43.0%) |
|  | No change | 56.8% (47.4%, 66.3%) | 62.4% (54.0%, 70.9%) | 58.8% (50.0%, 67.5%) | 52.4% (29.7%, 75.2%) | 23.6% (0.5%, 46.8%) | 46.9% (14.3%, 79.5%) |
|  | Slightly less | 13.8% (0.7%, 26.9%) | 13.2% (-1.5%, 27.8%) | 11.3% (-4.6%, 27.1%) | 2.5% (-10.7%, 15.6%) | 2.6% (-28.7%, 34.0%) | - |
|  | Much less | 15.0% (-0.9%, 30.8%) | 14.9% (-1.7%, 31.6%) | 23.6% (8.9%, 38.3%) | 21.1% (-15.1%, 57.3%) | 42.1% (-24.5%, 100.0%) | 49.0% (-34.6%, 100.0%) |
|  | Indicator of change | -14.5% (-34.4%, 6.3%) | -18.5% (-41.5%, 3.9%) | -28.4% (-51.9%, -5.1%) | 1.2% (-47.7%, 49.5%) | -12.9% (-100.0%, 75.8%) | -45.8% (-100.0%, 49.3%) |
| Ukraine | n | 478 | | | 173 | 53 | 23 |
|  | Much more | 3.7% (-13.2%, 20.6%) | 0.2% (-5.0%, 5.5%) | 1.3% (-14.3%, 16.8%) | 8.3% (-19.3%, 35.9%) | 11.9% (-31.0%, 54.8%) | - |
|  | Slightly more | 16.8% (0.9%, 32.7%) | 8.4% (-8.7%, 25.5%) | 9.9% (-8.1%, 27.8%) | 10.0% (-17.9%, 38.0%) | 24.2% (-15.3%, 63.8%) | 2.2% (-15.7%, 20.1%) |
|  | No change | 47.2% (34.2%, 60.3%) | 57.2% (45.7%, 68.8%) | 56.0% (44.4%, 67.7%) | 45.4% (21.9%, 69.0%) | 48.7% (15.2%, 82.2%) | 85.7% (58.3%, 100.0%) |
|  | Slightly less | 10.5% (-6.3%, 27.2%) | 12.6% (-4.8%, 29.9%) | 5.2% (-12.3%, 22.6%) | 13.9% (-16.9%, 44.7%) | 0.6% (-8.1%, 9.2%) | 11.8% (-49.7%, 73.3%) |
|  | Much less | 21.8% (5.2%, 38.3%) | 21.6% (4.8%, 38.4%) | 27.7% (11.3%, 44.0%) | 22.3% (-5.8%, 50.5%) | 14.6% (-28.5%, 57.7%) | 0.3% (-10.7%, 11.4%) |
|  | Indicator of change | -11.8% (-32.6%, 9.8%) | -25.5% (-48.2%, -3.1%) | -21.6% (-45.2%, 1.5%) | -17.3% (-54.5%, 19.4%) | 21.0% (-34.0%, 76.9%) | -10.4% (-74.1%, 54.5%) |
| United Kingdom | n | 920 | | | 182 | 66 | 60 |
|  | Much more | 27.4% (16.2%, 38.6%) | 17.4% (5.5%, 29.3%) | 17.0% (4.8%, 29.1%) | 17.8% (0.8%, 34.8%) | 29.2% (-11.2%, 69.7%) | 6.5% (-24.4%, 37.4%) |
|  | Slightly more | 28.1% (18.9%, 37.2%) | 25.6% (15.4%, 35.7%) | 21.2% (10.6%, 31.8%) | 23.6% (4.0%, 43.2%) | 23.1% (-4.3%, 50.5%) | 34.1% (-13.4%, 81.6%) |
|  | No change | 22.7% (12.4%, 32.9%) | 31.7% (22.4%, 41.1%) | 33.7% (24.5%, 42.8%) | 34.0% (11.7%, 56.2%) | 34.5% (-2.8%, 71.9%) | 33.3% (-6.0%, 72.7%) |
|  | Slightly less | 6.5% (-0.8%, 13.9%) | 10.5% (0.6%, 20.5%) | 11.0% (0.7%, 21.3%) | 10.1% (-15.4%, 35.5%) | 2.4% (-18.7%, 23.5%) | 3.7% (-12.9%, 20.2%) |
|  | Much less | 15.4% (4.6%, 26.1%) | 14.7% (4.8%, 24.7%) | 17.2% (7.5%, 26.8%) | 14.5% (-10.4%, 39.4%) | 10.8% (-14.0%, 35.5%) | 22.3% (4.7%, 39.9%) |
|  | Indicator of change | 33.5% (21.6%, 45.9%) | 17.8% (4.7%, 30.7%) | 10.1% (-3.6%, 23.3%) | 17.2% (-11.1%, 45.4%) | 39.3% (2.6%, 75.8%) | 14.3% (-31.8%, 60.7%) |
| Note. HED = Heavy episodic drinking, 95% CI = 95% confidence intervals. | | | | | | | |
